# Expression sequence tag library derived from peripheral blood mononuclear cells of the chlorocebus sabaeus

**DOI:** 10.1186/1471-2164-13-279

**Published:** 2012-06-22

**Authors:** Nicolas Tchitchek, Béatrice Jacquelin, Patrick Wincker, Carole Dossat, Corinne Da Silva, Jean Weissenbach, Antoine Blancher, Michaela Müller-Trutwin, Arndt Benecke

**Affiliations:** 1 Institut des Hautes Études Scientifiques - Centre National de la Recherche Scientifique, Bures-sur-Yvette, France; 2 Institut Pasteur, Unité de Régulation des Infections Rétrovirales, Paris, France; 3 CEA, Institut de Génomique, Genoscope, Evry, France; 4 Laboratoire d’Immunologie, CHU Rangueil, Toulouse, France; 5 Vaccine Research Institute, Institut Mondor de Recherche Biomédicale, INSERM U955, Créteil, France

## Abstract

**Background:**

African Green Monkeys (AGM) are amongst the most frequently used nonhuman primate models in clinical and biomedical research, nevertheless only few genomic resources exist for this species. Such information would be essential for the development of dedicated new generation technologies in fundamental and pre-clinical research using this model, and would deliver new insights into primate evolution.

**Results:**

We have exhaustively sequenced an Expression Sequence Tag (EST) library made from a pool of Peripheral Blood Mononuclear Cells from sixteen *Chlorocebus sabaeus* monkeys. Twelve of them were infected with the Simian Immunodeficiency Virus. The mononuclear cells were or not stimulated *in vitro* with Concanavalin A, with lipopolysacharrides, or through mixed lymphocyte reaction in order to generate a representative and broad library of expressed sequences in immune cells. We report here 37,787 sequences, which were assembled into 14,410 contigs representing an estimated 12% of the *C. sabaeus* transcriptome. Using data from primate genome databases, 9,029 assembled sequences from *C. sabaeus* could be annotated. Sequences have been systematically aligned with ten cDNA references of primate species including *Homo sapiens*, *Pan troglodytes*, and *Macaca mulatta* to identify ortholog transcripts. For 506 transcripts, sequences were quasi-complete. In addition, 6,576 transcript fragments are potentially specific to the *C. sabaeus* or corresponding to not yet described primate genes.

**Conclusions:**

The EST library we provide here will prove useful in gene annotation efforts for future sequencing of the African Green Monkey genomes. Furthermore, this library, which particularly well represents immunological and hematological gene expression, will be an important resource for the comparative analysis of gene expression in clinically relevant nonhuman primate and human research.

## Background

Nonhuman primates (NHP) are used in many areas of biomedical research because of their close relationship to humans. Indeed, for some human diseases, such as for HCV and HIV infections, they still represent the only available animal model. Moreover, optimal drug safety assessment and vaccine development are in many instances dependent on NHPs. Nowadays, the knowledge of their genome and transcriptome becomes critical for an efficient and parsimonious use of these models. The genome of the Chimpanzee (*Pan troglodytes*) [1], Indian rhesus macaque (Indian *Macaca mulatta*) [2], Orangutan (*Pongo abelii*[3], Chinese rhesus macaque (Chinese *Macaca mulatta*[4] and Cynomolgus macaque (*Macaca fascicularis*[4,5] have been sequenced, and sequencing of several other NHP genomes is ongoing [6,7]. The African Green Monkey (AGM) is a widely used species in biomedical research for studies in the field of immunology, neuroscience (such as Parkinson’s disease [8,9], cardiovascular disease [10], cell biology [11-13], pharmacology [14] and infectious diseases [15-19]. AGMs are one of the 40 natural hosts of the Simian Immunodeficiency Virus (SIV). They are particularly interesting models for studying of AIDS as this species is protected against the disease. Despite chronic infection by SIV, they generally do not develop any clinical symptoms [19,20] and hence are used to identify correlates of protection [19-21]. AGMs are divided into four species, named vervet (*Chlorocebus pygerythrus*), grivet (*Chlorocebus aethiops*), sabaeus (*Chlorocebus sabaeus*) and tantalus ( *Chlorocebus tantalus*). Among them, the vervet and sabaeus species have been most extensively studied [22-27]. Three hundred years ago, AGMs that belonged to the *C. sabaeus* species were transferred during slave trade from West/Central Africa to the Caribbean islands [28]. The only large breeding centers for AGMs are now located in these Islands, and the *C. sabaeus* species is now becoming the most studied AGM model for SIV and in biomedical research in general. In the context of viral infections such as with SIV, one of the main issues for the development of treatments and vaccines against human diseases, is to better understand the host transcriptomic responses of immune cells as the host immune response is mainly responsible for the outcome of the infection. Moreover, due to the important amount of genes expressed in case of activation, immune cells are relevant for revealing significant parts of the host transcriptome. So far, research involving AGMs, especially using gene expression profiling, were limited by the lack of sufficient gene sequence information and most studies were dependent on tools developed for human and more recently macaque species [7,29,30]. This limitation is a major problem since sequenced genes from AGMs revealed significant nucleotide differences from the human and even the macaque genomes [31-33], and more information on AGM gene sequences are therefore urgently needed. It should be noted however, that the difference between NHP and humans is higher at the level of which gene is expressed, rather than at the nucleotide diversity level [34]. In addition, it has been shown that NHP cells express additional genes that are not expressed in humans [35], and we have shown in a previous study that *C. sabaeus* express up to 16,000 genes in peripheral CD4+ cells, with 990 being specific of the species [36]. Annotating such sequences is challenging given that limited information is available and only few hundreds sequences of *C. sabaeus* are currently present in the GeneBank [37] database (Additional file 1: Figure S1). In this study, we constructed, sequenced and annotated a *C. sabaeus* EST (Expression Sequence Tag) library obtained from Peripheral Blood Mononuclear Cells (PBMC), as a tool for annotating AGM reference genomes, in order to allow the generation of technologies dedicated to analyze the immune responses in this species, as well as providing immediate valuable information to better understand the molecular and cellular mechanisms involved in AIDS resistance.

## Results

### Composition and assembly of the Chlorocebus sabaeus PBMC EST library

**Figure 1:**
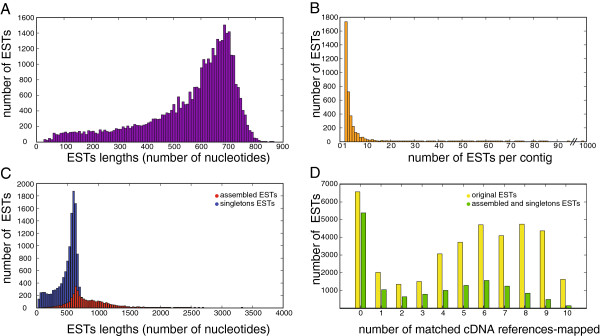
**Composition and alignment distribution of the EST library and the assembled distinct transcripts.** (**A**) Distribution of the length of the 37,787 original ESTs. The median EST length is equal to 618 nucleotides, the mean EST length is 563 nucleotides, and the standard deviation of the distribution is 167 nucleotides. (**B**) Distribution of the number of ESTs per contig in the ESTs assembly. The median number of ESTs per contig is equal to 3 ESTs, the mean number of ESTs per contig is 7 ESTs, and the standard deviation of the distribution is 27 ESTs. (**C**) Distribution of the length of the 14,410 distinct transcripts. The median sequence length is equal to 847 nucleotides, the mean sequence length is 943 nucleotides, and the standard deviation of the distribution is 388 nucleotides. The contribution of the assembled ESTs is shown in red while the contribution of singleton ESTs is shown in blue. (**D**) Distribution of the number of matched cDNA reference-mapped for both the 37,787 original ESTs (shown in yellow) and the 14,410 distinct transcripts (shown in green).

Our aim was to obtain the sequence information for the genes expressed in *C. sabaeus* immune cells. In order to be representative, we collected fresh PBMC from twelve SIV-infected and four non infected animals. In order to identify as many distinct transcripts as possible, we *in vitro* stimulated these cells or not with Concanavalin A (ConA), lipopolysaccharides (LPS) and by mixed lymphocyte reactions (MLR), as these stimuli upregulate mRNA expression of many genes. The different stimuli were chosen to activate distinct cellular receptors (T cell receptor, Toll-like receptors) and stimulate distinct immune cells (lymphocytes and antigen-presenting cells). Total RNA preparations from the stimulated and unstimulated cells were pooled and a cDNA library constructed. Sequences were obtained and sequence quality filtering showed that 37,787 ESTs were present in the library. They had a mean length of 563 nucleotides per EST with a standard deviation of 167 nucleotides (Figure 1A). The 37,787 ESTs have been assembled into 3,853 contigs (overlapping or embedded ESTs) and 10,557 singletons (not assembled ESTs). The median number of ESTs per contig was 3 with some outlier contigs being composed of up to 941 ESTs (Figure 1B). The mean length of the 14,410 assembled and singletons ESTs averages at 943 nucleotides (Figure 1C). The total length represented by our AGM EST library is about 21.10^6^ nucleotides and the total length of the assembled distinct transcripts 9.10^6^ nucleotides. Since the total length of the known *M. mulatta* distinct transcripts corresponds to 72.10^6^ nucleotides in the Ensembl database [38], our AGM sequences represent 12% of the *M. mulatta* transcriptome and potentialy a similar fraction of the AGM transcriptome.

### Inter- and intra- species comparisons

We then compared the ESTs to available transcriptomes of other primate species for annotation purposes and for quantification of transcript homologs. In order to get a general as well as a specific view, we used both the total 37,787 ESTs of the original library and the assembled distinct transcript library. They were aligned to available cDNA datasets of the following ten primate species: *Callithrix jacchus* (Ouistiti), *Gorilla gorilla* (Gorilla), *Homo sapiens* (Human), *M. mulatta*, *Microcebus murinus* (Mouse lemur), *Nomascus leucogeny* (Gibbon), *Otolemur garnettii* (Bushbaby), *P. troglodytes*, *P. abelii*, and *Tarsuis syrichta* (Tarsier) (Table 1). We applied stringent criteria for alignments. Thus, high-quality alignments have been filtered to only keep for each EST the best alignment for each species which maps at least 80% of the ESTs. 31,211 of the 37,787 total ESTs and 9,029 of the 14,410 assembled sequences could be aligned on cDNAs of at least one species. 29,191 of the total ESTs and 7,985 of the assembled ESTs have been aligned on at least two cDNA references. 1,628 of the total ESTs and 135 of the assembled ESTs have been aligned on all the 10 species, while 6,576 of the total ESTs and 5,384 of the assembled ESTs could not be mapped to any cDNA reference sets and are then potentially specific to the *C. sabaeus* transcriptome or highly diverse orthologs (Figure 1D). ESTs of the total and assembled AGM libraries have also been aligned to the draft assembly of the *M. fascicularis* genome [5] and a sequencing read library of the *C. sabaeus* genome [39]. Alignment results have been filtered to only keep for each EST the 5 best mapped reads when possible of the *C. sabaeus* draft scaffold genome, and the best mapped genomic position on the *M. fascicularis* draft assembly genome. Table 2 provides a summary of the results of the alignments with the 10 cDNA references and the 2 draft genomes. The highest number of aligned ESTs for both

**Table 1:** Composition details of the cDNA references

**Species name**	**Release version**	**Number of transcripts**	**Number of genes**
*C. jacchus*	Cjacchus3.2.1.63	55,137	32,339
*G. gorilla*	gorGor3.63	35,727	29,216
*H. sapiens*	GRCh37.63	174,598	53,894
*M. mulatta*	MMUL_1.63	44,725	30,247
*M. murinus*	micMur1.63	25,035	25,036
*N. leucogeny*	Nleu1.0.63	31,550	26,526
*O. garnettii*	BUSHBABY1.63	22,804	22,800
*P. troglodytes*	CHIMP2.1.63	41,488	27,116
*P. abelii*	PPYG2.63	31,566	28,088
*T. syrichta*	tarSyr1.63	20,261	20,215

For each species, the release version of the cDNA reference used and the number of transcripts and genes that composed the cDNA reference are indicated. All the cDNA references have been retrieved from the Ensembl [38] database.

**Table 2:** Alignment results of ESTs on the different cDNA references and genomes

**Species name**	**Target type**	**Original ESTs**			**Assembled and singleton ESTs**		
		**a.e.**	**m.t.**	**m.g.**	**a.e.**	**m.t.**	**m.g.**
*C. jacchus*	cDNA ref.	24,461 (64.73%)	5,951	5,051	5,954 (41.31%)	4,928	4,504
*G. gorilla*	cDNA ref.	23,633 (62.54%)	5,162	4,948	6,008 (41.69%)	4,622	4,527
*H. sapiens*	cDNA ref.	30,117 (79.70%)	9,208	6,529	8,708 (60.43%)	7,316	6,128
*M. mulatta*	cDNA ref.	24,213 (64.07%)	5,439	4,763	5,657 (39,25%)	4,585	4,273
*M. murinus*	cDNA ref.	8,618 (22.80%)	1,770	1,770	1,240 (08.60%)	1,138	1,138
*N. leucogeny*	cDNA ref.	22,600 (59.80%)	4,949	4,749	5,672 (39.36%)	4,389	4,296
*O. garnettii*	cDNA ref.	7,564 (20.01%)	1,431	1,431	930 (06.45%)	861	861
*P. troglodytes*	cDNA ref.	25,196 (66.67%)	5,699	5,156	6,332 (43.94%)	5,012	4,756
*P. abelii*	cDNA ref.	18,904 (50.02%)	4,149	3,989	4,274 (29.65%)	3,415	3,340
*T. syrichta*	cDNA ref.	5,327 (14.09%)	1,348	1,346	908 (06.30%)	854	854
*C. sabaeus*	d. scaf.	37,409 (98.99%)	–	–	14,139 (98,11%)	–	–
*M. fascicularis*	d. assem.	35,686 (94,44%)	–	–	13,392 (92.93%)	–	–

**Figure 2:**
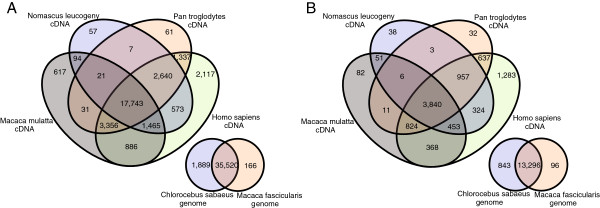
**Inter- and intra- species alignment comparisons.** (**A**) 4-set Venn diagram showing the intersections among the 4 sets of original ESTs aligned on the *H. sapiens*, *M. mulatta*, *P. troglodytes*, and *P. abelii* species, and 2-set Venn diagram showing the intersections between the 2 sets of original ESTs aligned over the *C. sabaeus* and *M. fascicularis* species. (**B**) Idem as A for the distinct transcripts.

For both the 37,787 originals ESTs and the 14,410 distinct transcripts, the number of aligned ESTs (a.e.) on the cDNA references (cDNA ref.), draft scaffold genome (d. scaf.), and draft assembly genome (d. assem.) are indicated. The number of mapped transcripts (m.t.) and mapped genes (m.g.) are also indicated for the cDNA references.

the original and the assembled ESTs was found for the *H. sapiens* (∼80% of the original ESTs and ∼60% of the distinct transcripts) probably due to the relatively higher degree of investigation of this genome. The higher frequence as to compared to the ones of NHP is thus due to the broader sequence information from human genomes and does not reflect the biological distances between the species. The *C. jacchus*, *G. gorilla*, *M. mulatta*, *N. leucogeny*, *P. troglodytes* species had relatively high-proportions of aligned ESTs (∼63% of the original ESTs and ∼41% of the distinct transcripts), and the *M. murinus*, *O. garnettii*, *T. syrichta* species had equally low-proportions of aligned ESTs (∼18% of the original library and ∼7% of the distinct transcripts). The *P. abelii* species had an intermediate proportion of aligned ESTs (∼50% of the library and ∼30% of the distinct transcripts). We then performed Venn Diagrams between AGM and cDNA of the NHP species showing the highest proportions of aligned ESTs (*M. mulatta*, *N. leucogeny*, *P. troglodytes*, and *H. sapiens*), 31,005 of the 37,787 original ESTs and 8,909 of the 14,410 distinct transcrips could been aligned on at least one of the cDNA references (Figures 2A and 2B). 23,450 of the original ESTs (62.05%) were shared between the *H. sapiens* and *M. mulatta* species. AGM shared 25,196 sequences (66.67%) with those of *M. mulatta*, and 17,743 (46.95%) with the four species. The number of mapped ESTs on the *C. sabaeus* and *M. fascicularis* draft genomes is highly significant for both the original ESTs and the assembled and singletons ESTs, and almost the totality of the ESTs are commonly mapped ESTs between the two genomes (Figures 2A and 2B). Note that the alignment to the draft genomes was performed using low-specificity alignment parameters and thus is not directly comparable to the alignments of the EST libraries. Overall, while giving different specific alignment information, the number of mapped transcripts and mapped genes for both the 37,787 originals ESTs and the 14,410 distinct transcripts are convergent in the number of mapped genes and proportional with the genomic distances that exist among these species.

### Specific comparison with the Macaca mulatta transcriptome

**Figure 3:**
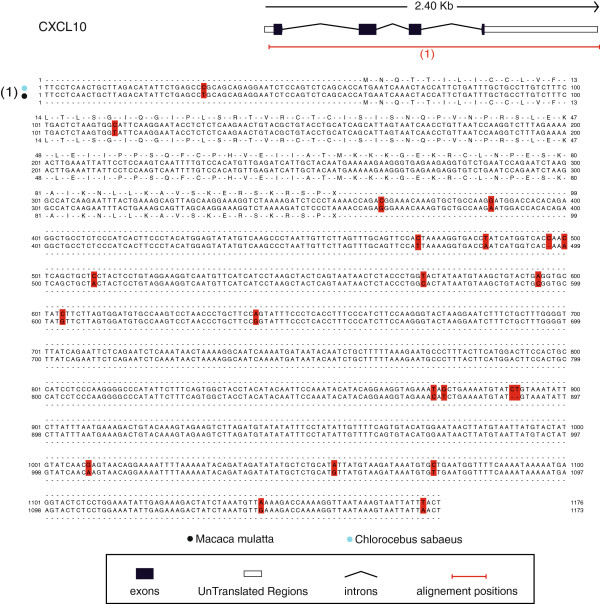
**Alignment details for the CXCL10 gene.** Alignment details for the C-X-C motif chemokine 10 gene of the *M. mulatta* species (Ensembl ID: ENSMMUT00000029391). Assembled ESTs have been aligned at different positions of the gene: (1) Contig2229.

The *M. mulatta* species is the closest primate species to the *C. sabaeus* for which significant genomic information is available. In order to gain additional information about the transcript fragments that we provide, we annotated them with the particular section positions of the messenger RNAs available for the *M. mulatta* species. We specified for each EST of the assembled library the positions of the 5’-untranslated region (5’UTR), coding DNA sequence (CDS), and 3’-untranslated region (3’UTR) based on the *M. mulatta* cDNA reference annotations. Among the 14,410 assembled ESTs, 11,211 could be annotated: 6,244 ESTs with the 5’UTR, 9,657 ESTs with the CDS, and 5,313 ESTs with the 3’UTR. We report 506 *M. mulatta* transcripts that have been mapped to more than 90% by an EST (Additional file 2: Table S1). CXCL10 (Figure 3) and S100A4 (Additional file 3: Figure S2) are part of these transcripts and given as examples.

### Quantification of expressed sequences and functional pathway analysis of the EST library

**Figure 4:**
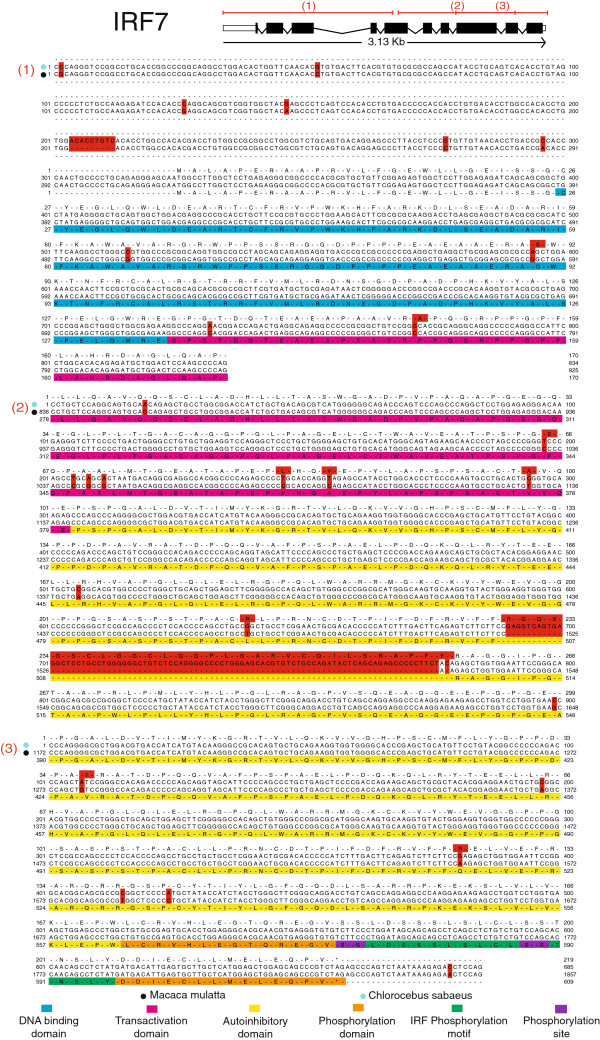
**Alignment details for the the IRF7 gene.** Alignment details for the Interferon regulatory factor 7 gene of the *M. mulatta* species (Ensembl ID: ENSMMUT00000009923). Assembled ESTs have been aligned at different positions of the gene: (1) Contig3553, (2) Contig866, (3) Contig1898. Same legend and nomenclature as in Figure 3.

**Figure 5:**
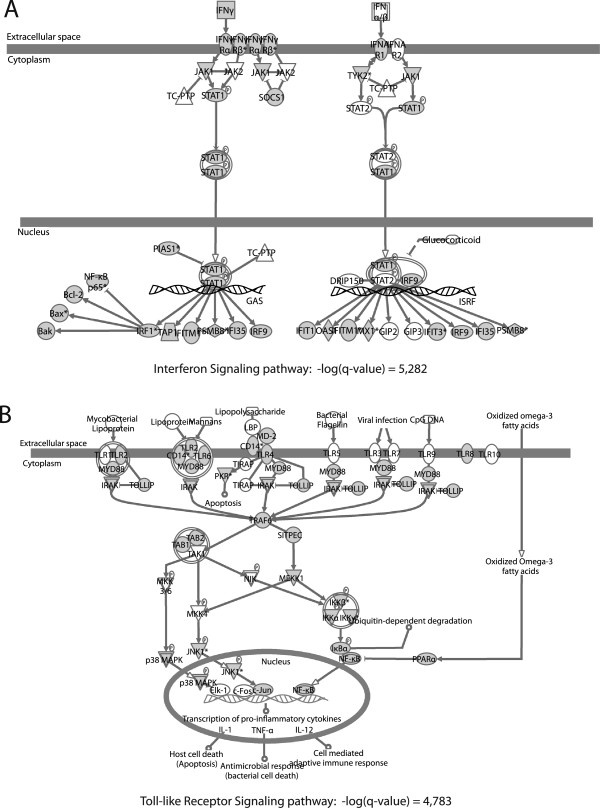
**Representation of the “Interferon Signaling” and “Toll-like Receptor Signaling” pathways.** (**A**) Representation of the “Interferon Signaling” pathway. (**B**) Representation of the “Toll-like Receptor Signaling” pathway. Genes present in the EST library are shown in gray.

In order to have a quantitative view of the expressed sequences of the *C. sabaeus* PBMC, we identified the most expressed transcripts in our EST library based on the *M. mulatta* homolog transcripts. Based on the 44,725 transcripts of the *M. mulatta* cDNA reference and the 14,410 ESTs of the original ESTs library, we calculated for each transcript the number of sequences mapped and obtained a list of the 50 most expressed *M. mulatta* ortholog transcripts in our EST library (Table 3). Among these most expressed transcripts, the hemoglobin beta (HBB) and alpha (HBA) genes were present, which might reflect a red blood cells contaminations of the PBMC, as well as more specific immune-related genes, such as CD74 and Granzyme B (GZMB). Some EST which correspond to genes which play an important role in immune responses against pathogens have been aligned: IRF7 (Figure 4), CD4 (Additional file 4: Figure S3), IFNG (Additional file 5: Figure S4), IFNGR1 (Additional file 6: Figure S5), IFNGR2 (Additional file 7: Figure S6). For all these transcripts, EST alignment positions as well as protein domains are given. Furthermore, in order to identify the over-represented pathways in our AGM EST library, we performed a functional canonical pathway analysis based on the list of the 9,208 *H. sapiens* transcripts uniquely mapped by the 37,787 original ESTs. Most of the canonical pathways found as statistically significantly over-represented are related to B and T cell signaling, and immune response pathways (Table 4). For instance, the “CD28 signaling in T Helper Cells”, “iCOS-iCOSL signaling in T Helper Cells”, “B Cell receptor Signaling” (Additional file 8: Figure S7A), and “T Cell receptor signaling” (Additional file 8: Figure S7B) pathways belong to the list of pathways found as significantly over-represented in our AGM library, as well as the “Glucocorticoid receptor signaling”, “Role of NFAT in regulation of the immune response” (Additional file 9: Figure S8A), “Antigen presentation pathway” (Additional file 9: Figure S8B), “JAK/STAT signaling”, and many different “Interleukin signaling” pathways. As a result of the *in vitro* stimulation of SIV-infected PBMC, the “NF-κB Activation by viruses” (Additional file 10: Figure S9A) and “Induction of apoptosis by HIV-1” (Additional file 10: Figure S9B) pathways are also significantly over-represented. Consistent with the stimulation by LPS, the “Interferon Signaling” (Figure 5A) and “Toll-like Receptor Signaling” (Figure 5B) pathways are also found significantly over-represented. Finally, ConA is capable of triggering positive selection in mature T cells by cross-linking the TCR with high avidity [40,41] and we found 8 pathways corresponding to these functions being induced (Table 4). The over-representation of gene transcripts belonging to these pathways of the immune system further indicates that this library is a valuable resource for profiling global gene expression in AGM immune cells. Overall, these gene and pathway information are consistent with what we could expect from an EST PBMC library.

**Table 3:** **List of the 50 most expressed *M. mulatta *ortholog transcripts in present EST library**

**Transcript ID**	**Gene symbol**	**Gene description**	**Count**
ENSMMUT00000006876	HBB_MACMU	Hemoglobin subunit beta	941
ENSMMUT00000045385	LOC712934		699
ENSMMUT00000012750	CD74		526
ENSMMUT00000015401	Q3YAP9_MACMU	eukaryotic translation elongation factor 1 alpha 1	519
ENSMMUT00000000859	HBA_MACMU	Hemoglobin subunit alpha	296
ENSMMUT00000017004	LOC712553		257
ENSMMUT00000038286		MTRNR2-like (LOC100499503)	232
ENSMMUT00000005322	B2MG_MACMU	Beta-2-microglobulin	212
ENSMMUT00000005104	LOC708526		208
ENSMMUT00000043999	RPL3	ribosomal protein L3	208
ENSMMUT00000038271	COX2_MACMU	Cytochrome c oxidase subunit 2	194
ENSMMUT00000027050	DRA_MACMU	Mamu class II histocompatibility antigen, DR alpha chain	191
ENSMMUT00000038268	Q6IYH3_MACMU	ATP synthase F0 subunit 6	185
ENSMMUT00000029930	Q3YAP9_MACMU	eukaryotic translation elongation factor 1 alpha 1	173
ENSMMUT00000045510	Q9GMG8_MACMU	acidic ribosomal phosphoprotein PO	173
ENSMMUT00000023666	LOC710590		155
ENSMMUT00000039116	LOC714576		144
ENSMMUT00000027943	B0Z9V5_MACMU	major histocompatibility complex, class I, E	143
ENSMMUT00000038267	Q6IYH2_MACMU	cytochrome c oxidase subunit III	135
ENSMMUT00000010560	B5MBT6_MACMU	ribosomal protein L13a	133
ENSMMUT00000032800	UBB	polyubiquitin-B	133
ENSMMUT00000010558	Q3YAQ2_MACMU	ribosomal protein S11	131
ENSMMUT00000011109		ribosomal protein S2 (RPS2)	131
ENSMMUT00000015005	LOC711043		129
ENSMMUT00000020179	GZMB		126
ENSMMUT00000033466	Q6IEB8_MACMU	interferon alpha-inducible protein 27	123
ENSMMUT00000038664	LOC719242		123
ENSMMUT00000008204	Q6IUG4_MACMU	glyceraldehyde-3-phosphate dehydrogenase	122
ENSMMUT00000029999	RPS20		116
ENSMMUT00000032342	TPT1		116
ENSMMUT00000012806	Q9GMG8_MACMU	acidic ribosomal phosphoprotein PO	115
ENSMMUT00000005819	SRGN		107
ENSMMUT00000040341	Q9MXS5_MACMU	MHC class I antigen	106
ENSMMUT00000014609	LOC711421		105
ENSMMUT00000004034	LOC710901		104
ENSMMUT00000009232	EEF1G	eukaryotic translation elongation factor 1 gamma	103
ENSMMUT00000027208	A2TJ58_MACMU	major histocompatibility complex, class II, DP alpha	94
ENSMMUT00000013155	Q6RHR8_MACMU	actin, cytoplasmic 1	93
ENSMMUT00000041082	E0WHM2_MACMU	MHC class I antigen	92
ENSMMUT00000043841	RPS3		89
ENSMMUT00000022628	A8QWZ5_MACMU	MHC class I antigen	86
ENSMMUT00000000617	RPL12	60S ribosomal protein L12	85
ENSMMUT00000018897	RPS6		84
ENSMMUT00000025324	ARHGDIB		79
ENSMMUT00000011502	A3F8W8_MACMU	MHC class II antigen	77
ENSMMUT00000040916	A3F8W8_MACMU	MHC class II antigen	76
ENSMMUT00000005540	LOC718964		75
ENSMMUT00000018430			75
ENSMMUT00000041189	A9XN15_MACMU	major histocompatibility complex, class I, A	73
ENSMMUT00000015586	Q6UIS1_MACMU	Actin beta subunit	72

For each of the 44,725 transcripts of the *M. mulatta* cDNA reference, we calculated the number of original ESTs mapped, and obtained a list of the 50 most expressed *M. mulatta* ortholog transcripts in our EST library. For each of the most expressed *M. mulatta* ortholog transcript, the Ensembl transcript ID, the gene symbol, the gene description, and the number of mapped ESTs is given.

**Table 4:** Top 50 canonical pathways found as significantly over-represented in present EST library

Canonical pathway	***−log*(q-value)**	***gen***_***a***_**/*gen***_***b***_
Protein Ubiquitination Pathway	16.80	148/274 (54%)
Glucocorticoid Receptor Signaling	16.80	148/295 (50%)
Oxidative Phosphorylation	15.00	92/159 (58%)
Mitochondrial Dysfunction	14.00	86/175 (49%)
CD28 Signaling in T Helper Cells	13.70	77/132 (58%)
Regulation of eIF4 and p70S6K Signaling	11.90	69/132 (52%)
Role of NFAT in Regulation of the Immune Response	10.70	97/200 (49%)
EIF2 Signaling	10.60	57/101 (56%)
PI3K/AKT Signaling	10.50	73/140 (52%)
iCOS-iCOSL Signaling in T Helper Cells	10.50	67/122 (55%)
B Cell Receptor Signaling	10.10	83/156 (53%)
Regulation of IL-2 Expression in Lymphocytes	9.70	53/89 (60%)
Integrin Signaling	9.48	104/209 (50%)
PKC*θ* Signaling in T Lymphocytes	8.93	68/142 (48%)
Hypoxia Signaling in the Cardiovascular System	8.93	46/68 (68%)
CTLA4 Signaling in Cytotoxic T Lymphocytes	8.52	57/98 (58%)
mTOR Signaling	8.51	79/162 (49%)
T Cell Receptor Signaling	8.43	59/109 (54%)
Type I Diabetes Mellitus Signaling	8.32	64/121 (53%)
Production of Nitric Oxide and ROS in Macrophages	8.32	83/187 (44%)
Ubiquinone Biosynthesis	8.05	45/112 (40%)
Molecular Mechanisms of Cancer	7.64	152/377 (40%)
Estrogen Receptor Signaling	7.54	70/136 (51%)
Antigen Presentation Pathway	7.20	30/43 (70%)
Apoptosis Signaling	7.19	53/96 (55%)
G2/M DNA Damage Checkpoint Regulation	7.19	32/49 (65%)
Prostate Cancer Signaling	6.96	49/97 (51%)
Phospholipase C Signaling	6.79	109/260 (42%)
Huntington’s Disease Signaling	6.78	104/238 (44%)
Chronic Myeloid Leukemia Signaling	6.65	54/105 (51%)
Pancreatic Adenocarcinoma Signaling	6.61	59/119 (50%)
IL-8 Signaling	6.60	86/193 (45%)
PI3K Signaling in B Lymphocytes	6.50	69/143 (48%)
Breast Cancer Regulation by Stathmin1	6.49	93/208 (45%)
IL-2 Signaling	6.31	35/58 (60%)
NF-*κ*B Activation by Viruses	6.26	44/82 (54%)
IL-15 Signaling	6.23	39/68 (57%)
T Helper Cell Differentiation	6.15	42/72 (58%)
TREM1 Signaling	6.05	35/66 (53%)
Fc*γ* Receptor-mediated Phagocytosis in Macrophages	5.98	52/102 (51%)
Pyrimidine Metabolism	5.85	70/213 (33%)
GM-CSF Signaling	5.69	38/67 (57%)
Induction of Apoptosis by HIV1	5.64	37/66 (56%)
Dendritic Cell Maturation	5.64	78/188 (41%)
NRF2-mediated Oxidative Stress Response	5.63	86/193 (45%)
Purine Metabolism	5.56	117/391 (30%)
fMLP Signaling in Neutrophils	5.50	57/128 (45%)
JAK/Stat Signaling	5.42	37/64 (58%)
HMGB1 Signaling	5.40	51/100 (51%)
IL-4 Signaling	5.40	40/73 (55%)

List of the top 50 canonical pathways found as statistically significantly over-represented in the functional pathway analysis of the EST library. For each canonical pathway, the associated multiple testing corrected p-value (shown as −*log*(q-value)) is indicated as well as the ratio between the number *gen*_*a *_of genes of the pathway mapped by the EST library and the total number *gen*_*b *_of genes defining the pathway.

### Analysis of the inter-species genomic relationships

**Figure 6:**
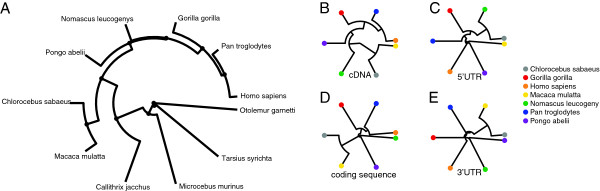
**Evolutionary relationships among primates species.** (**A**) phylogenetic tree of the 11 primate species for which the cDNA references were available calculated based on the 1,628 common original ESTs. (**B**) phylogenetic tree of the old world monkeys and human species calculated based on the 8,788 common assembled ESTs. (**C**) phylogenetic tree of the old world monkeys and human restricted to the 5’UTR of the transcripts calculated based on the 1,016 common assembled ESTs. (**D**) phylogenetic tree of the old world monkeys and human restricted to the coding sequence of the transcripts calculated based on the 8,024 common assembled ESTs. (**E**) phylogenetic tree of the old world monkeys and human restricted to the 3’UTR of the transcripts calculated based on the 2,209 common assembled ESTs.

Analysis of genomic relationships among species is an important way for studying evolution of genomic features. The relationships of the *C. sabaeus* EST with the 10 above described primate species for which the cDNA references were available have been quantified. For each one of the 1,628 ESTs aligned on all the cDNA references, multiple sequence alignment scores have been computed. Based on these pairwise alignment scores, an average genomic distance matrix has been computed (Table 5) and a phylogenetic tree constructed (Figure 6A). As it would be expected, the *H. sapiens* and *P. troglodytes* are clustered together, as it is also the case for the *C. sabaeus* and *M. mulatta*. The *G. gorilla*, *P. abelii*, and *N. leucogeny* were located between these two clusters, and the *C. jacchus*, *M. murinus*, *O. garnettii*, and *T. syrichta* are segregated from other species. By comparing only to the more related species, phylogenetic trees have also been computed with a higher number of AGM ESTs with all the 14,410 assembled transcripts (Figure 6B). Finally, trees have been constructed for specific sections of the transcripts: 5’UTR regions (Figure 6C), CDS sections (Figure 6D), and 3’UTR regions (Figure 6E). Both the phylogenetic trees restricted on the CDS and 3’UTR sections show a clusterisation of the *C. sabaeus* with the *M. mulatta* and a strong segregation with other species. Interestingly, the phylogenetic tree restricted on the 5’UTR sections revealed a different shape. *C. sabaeus* and the *N. leucogeny* species clustered together, suggesting distinct selective pressures in the 5’UTR as compared to other regions.

**Table 5:** Pairwise genomic distance matrix of the 11 primate species

	***C. sabaeus***	***G. gorilla***	***H. sapiens***	***M. mulatta***	***M. murinus***	***N. leucogeny***	***O. garnettii***	***P. troglodytes***	***P. abelii***	***T. syrichta***
*C. jacchus*	474	445	430	474	804	445	906	442	442	862
*C. sabaeus*		272	260	140	751	284	856	272	289	832
*G. gorilla*			103	263	741	191	858	117	200	830
*H. sapiens*				248	712	173	835	78	176	808
*M. mulatta*					754	266	873	259	290	842
*M. murinus*						753	770	734	743	897
*N. leucogeny*							876	185	224	842
*O. garnettii*								850	880	1006
*P. troglodytes*									191	824
*P. abelii*										859

Pairwise genomic distance matrix computed using the ESTs of the original library and the cDNA references of the 10 primates species for which the cDNA references were available. For pairs of species, the average multiple alignment score calculated over the 1,628 commonly aligned sequences is given. Scores have been rescaled by multiplication by 10^4^.

## Discussion

AGMs have provided useful animal models in biomedical research for many years [17,42-50]. They are also becoming a more and more essential model to the study of human biology and disease, such as neurological disorders [51,52] and AIDS [19,53]. Several studies could not be conducted so far because of the insufficiency of genomic resources on this primate [7]. This is a major limitation in view of the information that new generation technologies can offer for the progress in development of strategies to prevent or treat human diseases. The growing interest for this model is shown through the increase of the number of sequences published in the NCBI nucleotide database for this species every year (Additional file 1: Figure S1) and the sequencing of its genome which is underway. Nevertheless, our recent survey (as of January 11, 2012) showed that while there were 11,413,043 and 225,854 nucleotide sequences available for *H. sapiens* and *M. mulatta*, respectively, there were still only 2,527 nucleotide sequences for AGM in the databases. The primary goal of this study was to enhance the development of an AGM genomic resource through the construction, sequencing and characterization of a PBMC cDNA library of AGM (*C. sabaeus*). The results could be used to expand genomic research activities on this species.

We focused here on the construction of a cDNA library on blood immune cells (PBMC) in order to get as much immune defense genes as possible which could help to the study of several disease mechanisms such as the understanding of AIDS resistance in AGM. Therefore, to increase the expression of such genes, the cells were challenged or not with immune-relevant stimuli (ConA, LPS, MLR). We also chose to work on both, SIV-infected and non-infected animals, in order to eventually reveal new genes that might have a unique role in AIDS resistance in this natural host. The sequencing of the cDNAs yielded 37,787 ESTs with 14,410 assembled and singletons ESTs which cover 12% of the transcriptome. For annotation purpose, we aligned the 14,410 cDNA sequences of our library to the known cDNA libraries of 10 other primate species including the human one. Of the 31,005 ESTs identified, as many as 6,576 ESTs did not match any gene reported in the database. This high number of novel sequences might be due to the fact that the genomes of the other NHP species are not sufficiently annotated yet. However, a few of them might be true new gene candidates. Indeed, the stimulation used might have revealed a number of silent genes only expressed under the condition of infection.

As one would expect, at the CDS level, the divergence between *C. sabaeus* and *M. mulatta* was lower than between *C. sabaeus* and the other primate species. The gene distance at the non-coding regions was higher than in the CDS and higher for 5’UTR than 3’UTR. Interestingly, the 5’UTR of *C. sabaeus* did not cluster any more with *M. mulatta*, at least not consistently. This is in line with the fact that on average, 5’ and 3’ UTRs are less conserved across species than protein-coding sequences, with the 5’UTR being the most divergent, but still more conserved than untranscribed sequences [54,55]. It has been shown that high differences in the 5’UTR of orthologous genes correlate with their expression levels [56]. Indeed, this region is rich in regulatory elements. Changes in the regulation of gene expression levels play an important role in phenotypic diversity among closely related organisms [57,58]. The high distances observed at the 5’UTR region between the different primate species studied here might reflect part of these changes (Additional file 11: Table S2). However, we can not exclude that on one hand, our analyses might have misestimated the gene distance of UTR or ESTs in general, between the *C. sabaeus* and other species, as the length of the ESTs of our library are shorter (943 nucleotides) than the average length of human and macaque cDNAs (1,500 nucleotides) in the databases. On the other hand we might have overestimated this distance as compared to the rest of the transcriptome because the genes included in this library are mostly immune-related, thus among the most known divergent genes [59].

To further analyze the library, we determined the biological pathways represented by the 14,410 annotated ESTs. Among more general pathways (protein ubiquitination pathway, mitochondrial pathway), many pathways were related to the immune system (T cell activation, B cell activation) indicating the immune-specificity of the starting cells. The immune pathways appear well conserved in AGM, with most of the key components found in our library under the stimulation condition used (Figure 5 and Additional file 8: Figures S7, Additional file 9: Figures S8 and Additional file 10: Figures S9). Ubiquitously expressed genes, such as ribosomal proteins, housekeeping genes and mitochondrial pathway, are also included in this library and could be useful when using cell lines derived from AGM such as COS-7 and Vero cells. We studied in more detail genes which are of major importance for host immune defenses, such as IFN-*γ*, IFNGR, CXCL10 and IRF7 [60-62]. For the IFN-*γ*receptor (IFNGR), it has been shown in humans that any variation having a significant impact on IFNGR function is not tolerated [63]. Therefore, the deletion observed in the cytoplasmic tail of IFNGR1 in AGM as compared to macaque might either not have any functional consequence on this pathway or give to this species a yet unknown evolutionary advantage. Thus, it would be interesting to compare the sequence of AGM IFNGR1 with other SIV natural hosts in order to evaluate if this might play a role in AIDS resistance. CXCL10 (or IP-10) is a chemokine involved in the recruitment of cells of the immune system to sites of inflammation and is induced by IFN-*α* and IFN-*γ*[60]. Alteration of IP-10 expression has been associated with inflammatory diseases including infectious diseases, immune dysfunction and tumor development [64,65]. We did not find any difference at the amino acid level between the CXCL10 from *C. sabaeus* and the one from *M. mulatta*. This conservation suggests that any variation having an impact on CXCL10 function could be deleterious. IRF7 encodes a transcription factor which plays a role in the activation of virus-inducible cellular genes, including the type I interferon genes. The partial sequences from our library did not show the same mutations that were suggested to play a role in AIDS-resistance in another SIV-natural host, the sooty mangabey [66]. The mutations in IRF7 reported in one SM [66], were however also either not confirmed when studied in a large number of SM animals or found to be non-fixed and with no effects on the phenotype even when present in homozygosity (Johnson Z, Silvestri G, and Bosinger SE, personal communication). However, as this is not the same species, the mutations could be at other sites, or the mechanisms of AIDS resistance might be different between AGM and sooty mangabey. As our library was constructed on a pool of cells from 16 different animals, the sequences obtained are not representative of the inter-individual variability and need to be verified on the individual level for further studies.

To our knowledge, this is the first time that abundant genetic information on AGM is given. In this study, a total of 37,787 ESTs were sequenced, from which 14,410 contigs and singletons were identified, covering 12% of the AGM transcriptome. Moreover, this cDNA library provides both a large collection of novel transcripts and a detailed annotation of immune genes. The high volume of apparently novel AGM sequences suggests that our data could be a useful resource for future genomic investigation.

## Methods

### Construction and sequencing of the EST library

Twelve SIV-infected and four non-infected *C. sabaeus* (from Caribbean islands) were used in this study. The Central Committee for Animals at Institut Pasteur, Paris, France, reviewed and approved the use and care of animals. The experiments were performed according to national and European guidelines. Whole blood was collected from monkeys under anesthesia in heparinized tubes. PBMC were isolated from whole blood by density gradient centrifugation using the Lymphocyte Separation Medium 1077 (PAA Laboratories GmbH) and activated or not with different stimuli in RPMI-1640 with 10% fetal calf serum. For ConA activation (from Canavalia ensiformis (Sigma-Aldrich, St. Louis, MO, USA)): 4.10^6^ of isolated PBMC were plated with 10*μg.ml*^−1^ of ConA for 2, 6, 24, 36 or 72h. For LPS (E.Coli 0111:B4 Sigma (L2630)) activation: 4.10^6^ of isolated PBMC were plated with 10*μg.ml*^−1^ of LPS for 2, 6, 24, 36 or 72 hours. The MLR were done by mixing 4.10^6^ of isolated PBMC with 4.10^5^ PBMC from another animal for 2, 6, 24, 36 or 72h. Unstimulated cells were also kept for further RNA extraction. Total RNA was extracted from harvested cells by using the RNeasy®; Mini Kit (Qiagen, Courtaboeuf, France) following the manufacturer’s instructions. Briefly, cells were lysed in 350*μl* of RLT buffer, run over a QiaShredder column (Qiagen) to ensure homogeneous lysis, and resuspended in 30*μl* of sterile water. We added a DNase-RNase free (Qiagen) treatment on the column to eliminate any potential DNA contamination of RNA preparations. The quality and concentration of RNA was assessed as before [36]. The libraries were plated, arrayed robotically and bacterial clones have their plasmid DNA amplified using phi29 polymerase. The plasmids were end-sequenced by the Genoscope using BigDye Termination kits on Applied Biosystems 3730xl DNA Analysers.

### EST quality filtering

Poly-A and poly-T tails have been trimmed from the sequenced ESTs by using the trimest tool [67] (default parameters have been used) while starting and ending terminal N’s have been trimmed from the sequences using the trimseq tool [67] (a threshold cutoff parameter of 20% of Ns in a window of 30 nucleotides has been used).

### Assembly of the EST library

Assembly of ESTs into contigs has been performed using the EGassembler [68] tool. EGassembler aligns and merges sequence fragments resulting from shotgun sequencing or gene transcripts fragments in order to reconstruct the original segment or gene (an overlap identity cutoff parameter of 80% has been used).

### cDNA references and genomes used in this study

The *C. jacchus*, *G. gorilla*, *H. sapiens*, *M. mulatta*, *M. murinus*, *N. leucogeny*, *O. garnettii*, *P. troglodytes*, *P. abelii*, and *T. syrichta* cDNA references have been retrieved from the Ensembl [38] database. The sequencing of the *C. sabaeus* genome is currently in progress as part of an international collaborative effort at the Washington University Genome Center [39] and the draft scaffold genome release of this project has been used in this study. The draft assembly of the *M. fascicularis* genome used in this study is available through the ENA [69] database via accession numbers from FR874244 to FR874264 [5].

### ESTs alignment procedures

Alignment of the ESTs on the cDNA references and on the *M. fascicularis* draft assembly genome has been done using the BLAST tool [70] (an Expect value cutoff parameter of 10 has been used). Alignment results have been filtered to only keep for each EST the best alignment for each species that has at least a support of 80% with the EST sequence. Alignment of the ESTs on the *C. sabaeus* draft scaffold genome has been performed using the CBRC-LAST [71] based online tool available on the website of the Washington University Genome Center [72].

### Functional pathway analysis

The functional pathway analysis of the EST library has been performed using Ingenuity Pathways Analysis (IPA, Ingenuity®; Systems). IPA examines expressed genes in the context of known biological functions and pathways, mapping each gene identifier in a dataset to its corresponding molecule in the Ingenuity Pathways Knowledge Base (IPKB). P-values attributed to each pathway representing the statistical over-representation significance have been calculated by using the right-tailed Fisher’s exact test and have been adjusted using the Benjamini-Hochberg Multiple Testing correction [73]. Over the 9,208 *H. sapiens* transcripts uniquely mapped by the 37,787 original ESTs, 8,579 have been identified by the IPKB and then used in the functional analysis.

### Quantification of the evolutionary relationships and construction of the phylogenetic trees

Quantification of the evolutionary relationships among ESTs and EST mapped sequences has been performed using the Needleman-Wunsch multiple alignment algorithm [74]. Distance among sequences has been calculated using the Jukes-Cantor method [75] (maximum likelihood estimate) based on the NUC44 scoring matrix. Phylogenetic trees have been constructed by using the Unweighted Pair Group Method Average linking method (UPGMA, group average [76].

### Data accessibility

The 37,787 ESTs are available on the dbEST [77] database via the library entry named “*C. sabaeus* PBMC EST Library” (accession: LIBEST_027323) and via Accession Numbers from JK088433 to JK126219. Each EST entry has been annotated with its associated contig (for assembled ESTs), its best high-quality mapped transcript with the corresponding gene for each cDNA reference, its 5 best mapped reads (when available) on the *C. sabaeus* draft scaffold genome, and the genomic position of its best alignment on the *M. fascicularis* draft assembly genome.

## Competing interests

The authors declare no competing interests.

## Authors’ contributions

NT – bioinformatics and analysis of the library, writing of ms; BJ – cell isolation and library preparation, writing of ms; PW, CD, CDS, JW – library construction and sequencing; ABl – candidate gene analysis; MMT, ABe – project design, supervision, funding, writing of ms. All authors read and approved the final manuscript.

## Supplementary Material

Additional file 1 **Figure S1.** Number of AGM sequences published over the last years. Progression of AGM sequences published during the last two decades: this graph shows the number of AGM nucleotide sequences entered over each 5 year period in the NCBI nucleotide database with the number of sequences to be published in our EST library. Click here for file

Additional file 2 **Table S1.** List of the highly covered Macaca mulatta ortholog transcripts. List of the 506 *M. mulatta* ortholog transcripts that have been highly covered an assembled EST. For each *M. mulatta* transcript, the Ensembl transcript Id, the gene symbol, and the assembled EST that mapped the transcript at least at 90% are given. Click here for file

Additional file 3 **Figure S2.** Alignment details for the S100A4 gene. Alignment details for the S100 calcium binding protein A4 gene of the *M. mulatta* species (Ensembl ID: ENSMMUT00000015358). Assembled ESTs have been aligned at different positions of the gene: (1) Contig3147. Same legend and nomenclature as in Figure 3. Click here for file

Additional file 4 **Figure S3.** Alignment details for the CD4 gene. Alignment details for the CD4 gene of the *M. mulatta* species (Ensembl ID: ENSMMUT00000018518). Assembled ESTs have been aligned at different positions of the gene: (1) PP0ADA62YL02FM1. Same legend and nomenclature as in Figure 3. Click here for file

Additional file 5 **Figure S4.** Alignment details for the IFNG gene. Alignment details for the Interferon-gamma gene of the *M. mulatta* species (Ensembl ID: ENSMMUT00000027007). Assembled ESTs have been aligned at different positions of the gene: (1) Contig3283 (2) PP0ADA26YB24FM1. Same legend and nomenclature as in Figure 3. Click here for file

Additional file 6 **Figure S5.** Alignment details for the IFNGR1 gene. Alignment details for the Interferon Gamma Receptor 1 gene of the *M. mulatta* species (Ensembl ID: ENSMMUT00000016941). Assembled ESTs have been aligned at different positions of the gene: (1) Contig705 (2) PP0ADA55YK24FM1. Same legend and nomenclature as in Figure 3. Click here for file

Additional file 7 **Figure S6.** Alignment details for the IFNGR2 gene. Alignment details for the Interferon Gamma Receptor 2 gene of the *M. mulatta* species (Ensembl ID: ENSMMUG00000005508). Assembled ESTs have been aligned at different positions of the gene: (1) PP0ADA19YK11FM1. Same legend and nomenclature as in Figure 3. Click here for file

Additional file 8 **Figure S7.** Representation of the “B cell receptor signaling” and “T cell receptor signaling” pathways. (A) Representation of the “B cell receptor signaling” pathway. (B) Representation of the “T cell receptor signaling” pathway. Same legend and nomenclature as in Figure 5. Click here for file

Additional file 9 **Figure S8.** Representation of the “Role of NFAT in regulation of the immune response” and “Antigen presentation” pathways. (A) Representation of the “Role of NFAT in regulation of the immune response” pathway. (B) Representation of the “Antigen presentation” pathway. Same legend and nomenclature as in Figure 5. Click here for file

Additional file 10 **Figure S9.** Representation of the “NF-*κ*B activation by viruses” and “Induction of apoptosis by HIV-1” pathways. (A) Representation of the “NF-*κ*B activation by viruses” pathway. (B) Representation of the “Induction of apoptosis by HIV-1” pathway. Same legend and nomenclature as in Figure 5. Click here for file

Additional file 11 **Table S2.** Genomic distance matrix between the Chlorocebus sabaeus species and the old world monkeys and humans species. Genomic distance matrix computed between the ESTs of the original library and the mapped sequences of 7 old world monkey and human cDNA references restricted or not to specific regions of the transcripts (5’UTR, coding sequence, 3’UTR). For each comparing, the average multiple alignment score calculated over the commonly aligned sequences (c.a.s.) is given. Scores have been rescaled by multiplication by 10^4^. Click here for file
