# Supplementary figures and images for: Expression sequence tag library derived from peripheral blood mononuclear cells of the chlorocebus sabaeus
Source: BMC Genomics. 2012 Jun 22;13:279. doi: 10.1186/1471-2164-13-279 (PMC3539953; doi:10.1186/1471-2164-13-279)

# Supplementary Figure 1

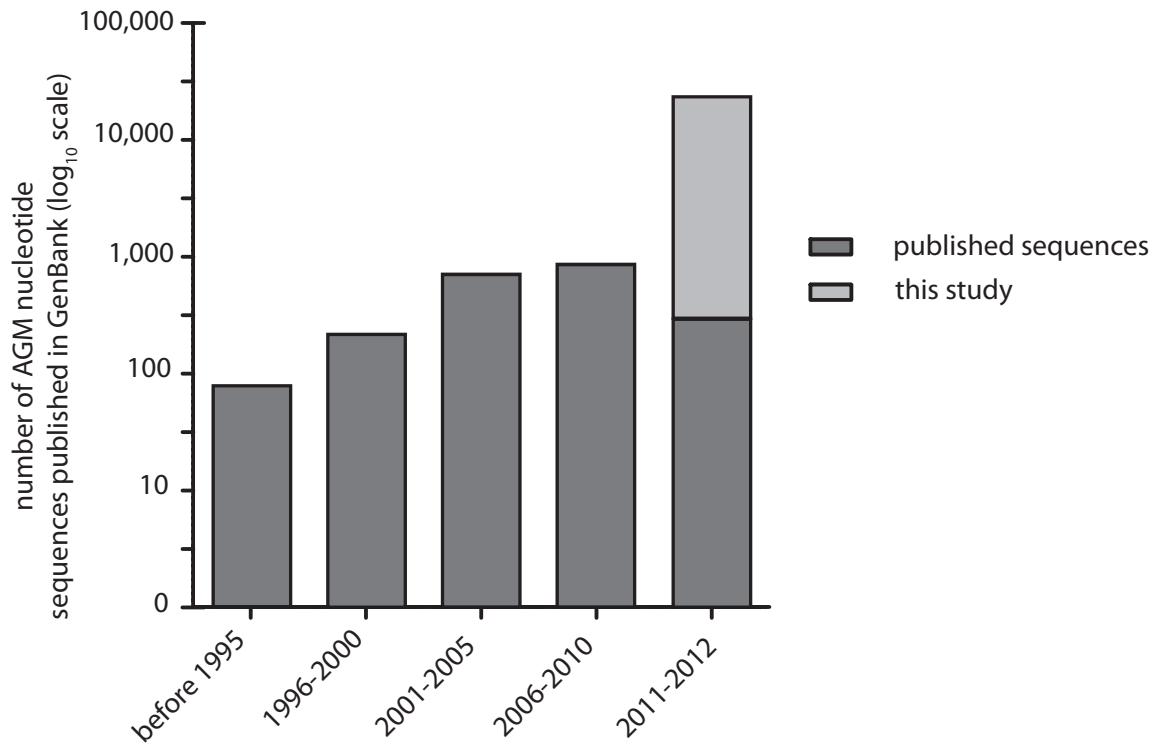

Supplement: Additional file 1 — Figure S1. Number of AGM sequences published over the last years. Progression of AGM sequences published during the last two decades: this graph shows the number of AGM nucleotide sequences entered over each 5 year period in the NCBI nucleotide database with the number of sequences to be published in our EST library. [file 1471-2164-13-279-S1.pdf]

# Supplementary Figure 3

CD4

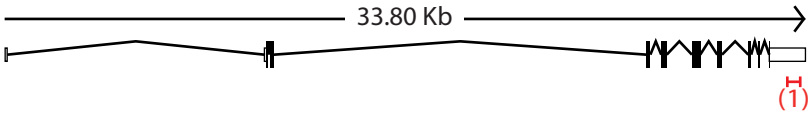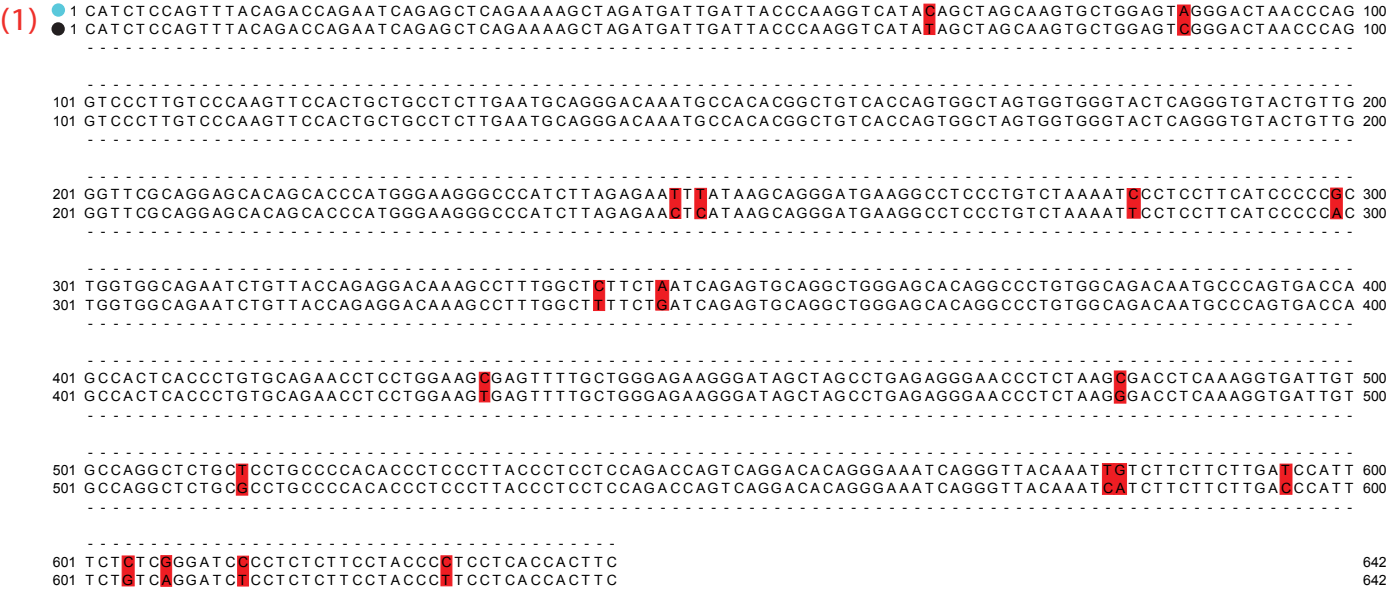

Supplement: Additional file 4 — Figure S3. Alignment details for the CD4 gene. Alignment details for the CD4 gene of the M. mulatta species (Ensembl ID: ENSMMUT00000018518). Assembled ESTs have been aligned at different positions of the gene: (1) PP0ADA62YL02FM1. Same legend and nomenclature as in Figure 3. [file 1471-2164-13-279-S4.pdf]

# Supplementary Figure 6

## IFNGR2

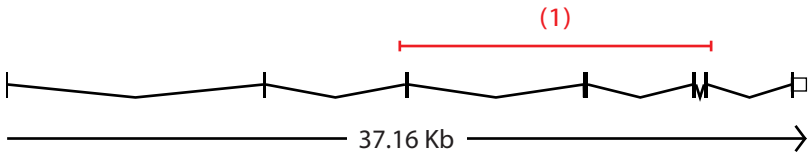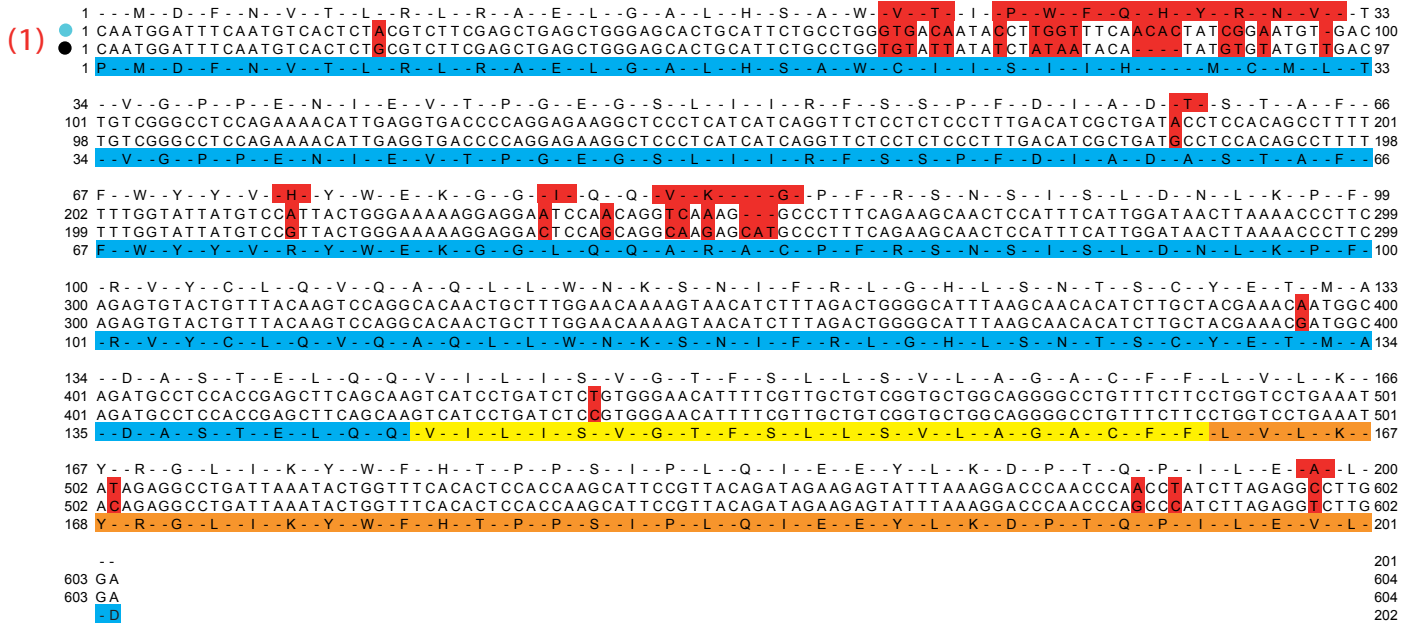

● Macaca mulatta

● Chlorocebus sabaeus

Supplement: Additional file 7 — Figure S6. Alignment details for the IFNGR2 gene. Alignment details for the Interferon Gamma Receptor 2 gene of the M. mulatta species (Ensembl ID: ENSMMUG00000005508). Assembled ESTs have been aligned at different positions of the gene: (1) PP0ADA19YK11FM1. Same legend and nomenclature as in Figure 3. [file 1471-2164-13-279-S7.pdf]
