# Supplementary material for: Expression sequence tag library derived from peripheral blood mononuclear cells of the chlorocebus sabaeus
Source: BMC Genomics. 2012 Jun 22;13:279. doi: 10.1186/1471-2164-13-279 (PMC3539953; doi:10.1186/1471-2164-13-279)
Supplement: Additional file 2 — Table S1. List of the highly covered Macaca mulatta ortholog transcripts. List of the 506 M. mulatta ortholog transcripts that have been highly covered an assembled EST. For each M. mulatta transcript, the Ensembl transcript Id, the gene symbol, and the assembled EST that mapped the transcript at least at 90% are given. [file 1471-2164-13-279-S2.pdf]

Supplementary Table 1

List of the highly covered Macaca mulatta ortholog transcripts.

Transcript ID	Gene Symbol	EST ID
ENSMMUT00000000038	CKLF	PP0ADA13YG07FM1
ENSMMUT00000000232	LOC719555	Contig3818
ENSMMUT00000000333	GLHA_MACMU	PP0ADA2YD10FM1
ENSMMUT00000000333	GLHA_MACMU	PP0ADA59YE03FM1
ENSMMUT00000000386	SNRPD3	Contig580
ENSMMUT00000000389	RPS10	PP0ADA36YE10FM1
ENSMMUT00000000414	Q4G3W7_MACMU	Contig1508
ENSMMUT00000000422	CCL2_MACMU	Contig512
ENSMMUT00000000431	LOC710859	Contig2840
ENSMMUT00000000526	TOMM22	Contig202
ENSMMUT00000000526	TOMM22	PP0ADA50YP08FM1
ENSMMUT00000000617	RPL12	Contig754
ENSMMUT00000000814	DGCR6	Contig2246
ENSMMUT00000000954	GBP1	Contig3034
ENSMMUT00000000988	LOC710486	PP0ADA101YC10FM1
ENSMMUT00000001068	CCDC127	PP0ADA5YD10FM1
ENSMMUT00000001175	CD53	Contig646
ENSMMUT00000001196	DAD1	PP0ADA69YK14FM1
ENSMMUT00000001240	LOC710189	Contig35
ENSMMUT00000001296	SUCLG1	Contig2826
ENSMMUT00000001343	CCL3_MACMU	PP0ADA106YD14FM1
ENSMMUT00000001389	LOC712622	Contig393
ENSMMUT00000001449	ABC3H_MACMU	Contig173
ENSMMUT00000001693	LOC702360	Contig2874
ENSMMUT00000001818	SF3B14	Contig2070
ENSMMUT00000001878	TIMM10	PP0ADA91YE20FM1
ENSMMUT00000001896	RPL32	Contig3737
ENSMMUT00000001913	LOC721592	PP0ADA61YA10FM1
ENSMMUT00000002058	ARPC1B	Contig2419
ENSMMUT00000002059	LOC707779	Contig1039
ENSMMUT00000002126	PSMD6	Contig2026
ENSMMUT00000002157	SLC25A26	Contig2323
ENSMMUT00000002177	A4Q982_MACMU	Contig2863
ENSMMUT00000002224	TTC32	Contig413
ENSMMUT00000002226	LOC708475	Contig2525
ENSMMUT00000002252	LOC713428	PP0ADA82YJ13FM1
ENSMMUT00000002260	GZMA	Contig993
ENSMMUT00000002326		Contig2656
ENSMMUT00000002371	SSNA1	Contig638
ENSMMUT00000002383	Q3YAI7_MACMU	Contig1037
ENSMMUT00000002415	SODC_MACMU	Contig1710
ENSMMUT00000002615	XRCC6	Contig2878
ENSMMUT00000002683	MRPS31	Contig944
ENSMMUT00000002844	LOC719886	Contig3636

ENSMMUT00000002858		Contig3111
ENSMMUT00000002872	MYST1	PP0ADA87YH09FM1
ENSMMUT00000003076	LOC717349	PP0ADA46YF16FM1
ENSMMUT00000003109	LOC717640	Contig3333
ENSMMUT00000003388	PSENEN	Contig366
ENSMMUT00000003393	PSENEN	Contig2814
ENSMMUT00000003486		Contig2152
ENSMMUT00000003491	RRP36	Contig1896
ENSMMUT00000003706	Q9N1D9_MACMU	Contig1068
ENSMMUT00000003883	LOC709107	Contig2270
ENSMMUT00000003961	ARF1	Contig2720
ENSMMUT00000004034	LOC710901	Contig1640
ENSMMUT00000004187	LOC722319	PP0ADA57YP01FM1
ENSMMUT00000004252	CUEDC2	Contig286
ENSMMUT00000004274	B3Y6B1_MACMU	PP0ADA8YM15FM1
ENSMMUT00000004280	CCT8	Contig3495
ENSMMUT00000004290	LOC719419	Contig3323
ENSMMUT00000004299	ACAA2	Contig2066
ENSMMUT00000004310	LOC721259	PP0ADA80YB17FM1
ENSMMUT00000004316	LOC707241	Contig592
ENSMMUT00000004399		Contig377
ENSMMUT00000004401	CDC37	Contig2337
ENSMMUT00000004489	CCT4	Contig2349
ENSMMUT00000004652	B8YE08_MACMU	Contig2888
ENSMMUT00000004666	TUFM	Contig1013
ENSMMUT00000004684	ATP5F1	Contig3548
ENSMMUT00000004754	BCL2A1	Contig1028
ENSMMUT00000004807	BIRC3	Contig943
ENSMMUT00000004870	TMSB10	Contig579
ENSMMUT00000004937	LOC712754	Contig2843
ENSMMUT00000004994	CD8B	Contig3804
ENSMMUT00000005013	RPUSD3	Contig3667
ENSMMUT00000005045	ATP5A1	Contig3220
ENSMMUT00000005079	EXOSC8	Contig176
ENSMMUT00000005400	NOSIP	Contig1271
ENSMMUT00000005429	BATF	Contig1406
ENSMMUT00000005485	PFDN6	Contig79
ENSMMUT00000005540	LOC718964	Contig2022
ENSMMUT00000005611	RHOG	Contig519
ENSMMUT00000005657	LOC698965	Contig3808
ENSMMUT00000005861	SHFM1	Contig2786
ENSMMUT00000005927	LOC722173	Contig1431
ENSMMUT00000006217	CIB1	Contig277
ENSMMUT00000006239	RAD23A	Contig2596
ENSMMUT00000006254	PGPEP1	PP0ADA50YB10FM1
ENSMMUT00000006296	LOC717324	Contig215
ENSMMUT00000006303	LOC100430880	PP0ADA18YE02FM1
ENSMMUT00000006306	MAT2A	Contig3341
ENSMMUT00000006333	ZNF576	Contig1862
ENSMMUT00000006375	ATF4	Contig1856
ENSMMUT00000006379	RPL23	Contig2943
ENSMMUT00000006395	LOC720291	Contig1551
ENSMMUT00000006404	POLD4	Contig2484
ENSMMUT00000006407	PPBP	Contig2859
ENSMMUT00000006633	HSPA8	Contig1931
ENSMMUT00000006645	LOC719527	Contig1422

ENSMMUT00000006736	TIFAB	PP0ADA77YB21FM1
ENSMMUT00000006837	C3H7orf30	Contig783
ENSMMUT00000006869	NDUFS4	PP0ADA83YJ01FM1
ENSMMUT00000006878	HBB_MACMU	Contig383
ENSMMUT00000006892	HNRNPL	Contig1161
ENSMMUT00000007058	RPS21	Contig1997
ENSMMUT00000007122	EDF1	Contig1316
ENSMMUT00000007191	PEBP1	Contig380
ENSMMUT00000007265	MRPS25	PP0ADA70YM04FM1
ENSMMUT00000007448	LOC715807	Contig3055
ENSMMUT00000007765		Contig1137
ENSMMUT00000007765		Contig1628
ENSMMUT00000007765		PP0ADA32YP18FM1
ENSMMUT00000007765		PP0ADA44YE13FM1
ENSMMUT00000007812	MED29	Contig1059
ENSMMUT00000007833	AL5AP_MACMU	Contig2889
ENSMMUT00000008089	LOC718643	Contig199
ENSMMUT00000008106	LOC722178	PP0ADA72YL06FM1
ENSMMUT00000008148	PSMA5	Contig1062
ENSMMUT00000008204	Q6IUG4_MACMU	Contig1830
ENSMMUT00000008401	Q4G3Y5_MACMU	Contig1641
ENSMMUT00000008415	Q0PDN4_MACMU	Contig3101
ENSMMUT00000008462	TMED9	Contig421
ENSMMUT00000008600		PP0ADA53YL17FM1
ENSMMUT00000008622	MDH1	Contig679
ENSMMUT00000008701	LOC706142	Contig2773
ENSMMUT00000008748	LOC717900	PP0ADA55YG16FM1
ENSMMUT00000008775	CSTA	PP0ADA58YI13FM1
ENSMMUT00000008776	AMICA1	Contig3287
ENSMMUT00000008847	LOC716997	Contig2379
ENSMMUT00000008922	C7H14orf147	Contig3798
ENSMMUT00000009101	ARPC3	Contig736
ENSMMUT00000009144	RT18B_MACMU	Contig3765
ENSMMUT00000009187	AP2M1	Contig3264
ENSMMUT00000009232	EEF1G	Contig1560
ENSMMUT00000009286	LOC710521	Contig2811
ENSMMUT00000009372	C16orf91	Contig2160
ENSMMUT00000009459	UBE2G2	PP0ADA46YH13FM1
ENSMMUT00000009465	COX4I1	Contig2077
ENSMMUT00000009465	COX4I1	PP0ADA37YH01FM1
ENSMMUT00000009517	G6PC3	Contig1623
ENSMMUT00000009535	LOC695847	PP0ADA5YB14FM1
ENSMMUT00000009602	S100A9	Contig932
ENSMMUT00000009698	SNRPD2	Contig3254
ENSMMUT00000009754	Q4G406_MACMU	Contig2833
ENSMMUT00000009775	LOC100430606	PP0ADA74YI15FM1
ENSMMUT00000009787	RASSF1	Contig1886
ENSMMUT00000009800	CD164	Contig2547
ENSMMUT00000009990	LOC699632	PP0ADA7YH10FM1
ENSMMUT00000010239	CORO1A	Contig682
ENSMMUT00000010246		PP0ADA15YB02FM1
ENSMMUT00000010461	DHRS4	Contig2571
ENSMMUT00000010558	Q3YAQ2_MACMU	Contig961
ENSMMUT00000010558	Q3YAQ2_MACMU	PP0ADA1YB18FM1
ENSMMUT00000010560	B5MBT6_MACMU	Contig674
ENSMMUT00000010569		PP0ADA78YK21FM1

ENSMMUT00000010651	LOC714261	Contig439
ENSMMUT00000011086	ZNHI3_MACMU	Contig2144
ENSMMUT00000011109		Contig515
ENSMMUT00000011121	LOC717749	Contig3158
ENSMMUT00000011164	PSMD4	Contig2446
ENSMMUT00000011192	TYOBP_MACMU	Contig1373
ENSMMUT00000011307	LOC712352	Contig2364
ENSMMUT00000011322	IRF1	Contig1015
ENSMMUT00000011476	IGKC	Contig1619
ENSMMUT00000011502	A3F8W8_MACMU	Contig1605
ENSMMUT00000011586	LOC693645	Contig2902
ENSMMUT00000011699	LOC716898	Contig1077
ENSMMUT00000011762	LOC702933	PP0ADA70YI17FM1
ENSMMUT00000011786	ALAS2	Contig2808
ENSMMUT00000012069		PP0ADA5YE07FM1
ENSMMUT00000012100	DRAP1	Contig1355
ENSMMUT00000012136	NOL7	Contig1882
ENSMMUT00000012165		PP0ADA49YF14FM1
ENSMMUT00000012197	LOC711259	Contig1192
ENSMMUT00000012227	RPL26	Contig942
ENSMMUT00000012334	LOC714727	Contig3480
ENSMMUT00000012374	LST1_MACMU	Contig1682
ENSMMUT00000012613	EIF3M	Contig1815
ENSMMUT00000012750	CD74	Contig2792
ENSMMUT00000012806	Q9GMG8_MACMU	Contig2044
ENSMMUT00000012863	CYTC_MACMU	Contig691
ENSMMUT00000012874	LOC713768	Contig2424
ENSMMUT00000012932	LOC709646	Contig3597
ENSMMUT00000012942		PP0ADA69YI09FM1
ENSMMUT00000012994	HSD17B10	Contig2181
ENSMMUT00000013043	HSD17B7	PP0ADA73YK02FM1
ENSMMUT00000013168	SF3B5	Contig1218
ENSMMUT00000013205	Q4G3Z7_MACMU	Contig1233
ENSMMUT00000013323	LOC708539	Contig2493
ENSMMUT00000013333	LOC710112	Contig3261
ENSMMUT00000013384	EIF4A3	Contig2806
ENSMMUT00000013393	C2orf79	PP0ADA108YA05FM1
ENSMMUT00000013435		PP0ADA90YL08FM1
ENSMMUT00000013614	LOC708821	Contig2970
ENSMMUT00000013766	CASP1	Contig181
ENSMMUT00000013849	LOC719983	Contig3380
ENSMMUT00000013876	ZBTB8OS	PP0ADA71YB22FM1
ENSMMUT00000013943	HINT1	Contig477
ENSMMUT00000013975	LOC697736	Contig2707
ENSMMUT00000013975	LOC697736	PP0ADA68YC17FM1
ENSMMUT00000014001		Contig2598
ENSMMUT00000014035	SNRPB	Contig247
ENSMMUT00000014086		PP0ADA91YB22FM1
ENSMMUT00000014244	C12orf57	Contig3179
ENSMMUT00000014246	PTPN6	Contig1237
ENSMMUT00000014262	GLIPR2	Contig2051
ENSMMUT00000014314	CD52	Contig1064
ENSMMUT00000014758	LOC721795	PP0ADA106YK04FM1
ENSMMUT00000014909	SEC61B	Contig3513
ENSMMUT00000015005	LOC711043	Contig2316
ENSMMUT00000015072	C7orf23	Contig3537

ENSMMUT00000015127	LOC716495	Contig3477
ENSMMUT00000015131	CCT2	Contig472
ENSMMUT00000015137	LOC716529	PP0ADA67YA12FM1
ENSMMUT00000015358	S100A4	Contig3147
ENSMMUT00000015401	Q3YAP9_MACMU	Contig1180
ENSMMUT00000015469	LOC706751	PP0ADA41YC23FM1
ENSMMUT00000015500	COX16	PP0ADA8YK15FM1
ENSMMUT00000015551	Q3I226_MACMU	Contig1494
ENSMMUT00000015586	Q6UIS1_MACMU	Contig457
ENSMMUT00000015816	TAP1	Contig1593
ENSMMUT00000015819	LOC717766	Contig2578
ENSMMUT00000015822	PSMB9	Contig2406
ENSMMUT00000015874	LOC698069	Contig1344
ENSMMUT00000015906	EIF3E	Contig3689
ENSMMUT00000015919	DNAJC30	PP0ADA68YA10FM1
ENSMMUT00000016036	Q6IEA0_MACMU	Contig3635
ENSMMUT00000016218	LOC719249	Contig1398
ENSMMUT00000016554	AIP	Contig341
ENSMMUT00000016567	SNRPA1	Contig1116
ENSMMUT00000016603	LOC712612	PP0ADA19YF19FM1
ENSMMUT00000016863	CYB5B	PP0ADA43YN22FM1
ENSMMUT00000017095	MLF2	Contig3828
ENSMMUT00000017179	B3Y667_MACMU	Contig1504
ENSMMUT00000017192	Q6SZ60_MACMU	Contig805
ENSMMUT00000017336	PSMB3	PP0ADA22YN12FM1
ENSMMUT00000017570	LOC697324	Contig3049
ENSMMUT00000017571	C19H19orf59	PP0ADA95YB08FM1
ENSMMUT00000017765	ANXA1	Contig2031
ENSMMUT00000017980		PP0ADA65YM15FM1
ENSMMUT00000017980		PP0ADA92YP18FM1
ENSMMUT00000018108	YBEY	PP0ADA16YL23FM1
ENSMMUT00000018147	LOC717864	Contig542
ENSMMUT00000018227	VMO1	Contig1189
ENSMMUT00000018233	Q3YAI3_MACMU	Contig2099
ENSMMUT00000018497	Q6VEU4_MACMU	Contig3233
ENSMMUT00000018577	Q3YAJ0_MACMU	Contig2565
ENSMMUT00000018596	MFF	Contig257
ENSMMUT00000018609	IFITM3	PP0ADA107YG16FM1
ENSMMUT00000018609	IFITM3	PP0ADA80YD18FM1
ENSMMUT00000018610	IFITM3	Contig978
ENSMMUT00000018615	PCNA	Contig24
ENSMMUT00000018771	LOC708535	Contig2540
ENSMMUT00000018787	Q4G3W7_MACMU	PP0ADA83YF10FM1
ENSMMUT00000018838	LOC706281	Contig3618
ENSMMUT00000018858	MCM7	Contig1992
ENSMMUT00000018897	RPS6	Contig1127
ENSMMUT00000019009	IL27RA	Contig3630
ENSMMUT00000019182	LOC712367	PP0ADA55YJ08FM1
ENSMMUT00000019309	LOC694957	Contig3825
ENSMMUT00000019408	ATP6V0E1	Contig1168
ENSMMUT00000019411	SPC25	Contig3116
ENSMMUT00000019704	LOC100428450	Contig196
ENSMMUT00000019756	Q5TM71_MACMU	Contig1122
ENSMMUT00000019781		Contig2277
ENSMMUT00000019938	TNFRSF18	Contig1353
ENSMMUT00000020179	GZMB	Contig795

ENSMMUT00000020201	NINJ1	Contig2037
ENSMMUT00000020582	LOC718240	Contig1080
ENSMMUT00000020744	CD37	Contig3458
ENSMMUT00000020889	RPLP1	Contig822
ENSMMUT00000021088	LOC701909	PP0ADA91YL19FM1
ENSMMUT00000021105	RPL24	Contig324
ENSMMUT00000021247	ATP6V0B	Contig3650
ENSMMUT00000021339	NOL12	Contig2677
ENSMMUT00000021409	LOC703957	PP0ADA69YN14FM1
ENSMMUT00000021418	CHCHD2	Contig555
ENSMMUT00000021423	SDHAF2	Contig2149
ENSMMUT00000021583	PDCD5	Contig105
ENSMMUT00000021628	LOC699426	Contig3784
ENSMMUT00000021634		Contig3508
ENSMMUT00000021656	SAMM50	Contig940
ENSMMUT00000021743	ATP5O	Contig1925
ENSMMUT00000021829	RPS27A	Contig2676
ENSMMUT00000021882	BLVRB	PP0ADA94YA12FM1
ENSMMUT00000022083	MAMU-DOA	Contig2768
ENSMMUT00000022115	MAGMAS	PP0ADA15YB18FM1
ENSMMUT00000022211	LOC710837	Contig111
ENSMMUT00000022291	LOC706099	Contig557
ENSMMUT00000022352	LOC718783	Contig313
ENSMMUT00000022572	LOC100428826	Contig1287
ENSMMUT00000022622	Q3YAI8_MACMU	Contig2003
ENSMMUT00000022815	MRPS5	Contig2498
ENSMMUT00000023092	PSMA1	Contig114
ENSMMUT00000023234	LOC702831	Contig1937
ENSMMUT00000023236	PSMA6	Contig126
ENSMMUT00000023299	MRPL23	PP0ADA20YG20FM1
ENSMMUT00000023348	SERPINB2	Contig593
ENSMMUT00000023359	C9orf123	Contig3354
ENSMMUT00000023383	PPIA_MACMU	Contig1415
ENSMMUT00000023420	LOC711541	Contig249
ENSMMUT00000023420	LOC711541	PP0ADA17YB19FM1
ENSMMUT00000023666	LOC710590	Contig1591
ENSMMUT00000023923	Q8WMJ2_MACMU	Contig3306
ENSMMUT00000023953	MRPL18	Contig138
ENSMMUT00000024077	ISCU	Contig266
ENSMMUT00000024087	C12orf45	PP0ADA84YD06FM1
ENSMMUT00000024099	CDC123	Contig1391
ENSMMUT00000024125	LOC716166	Contig3833
ENSMMUT00000024125	LOC716166	PP0ADA46YM20FM1
ENSMMUT00000024201	EIF3G	Contig146
ENSMMUT00000024220		PP0ADA80YA14FM1
ENSMMUT00000024235	GPR171	Contig2964
ENSMMUT00000024428	SPCS1	Contig2552
ENSMMUT00000024507	RABAC1	Contig2374
ENSMMUT00000024761	CTSL	Contig3586
ENSMMUT00000024879		Contig2280
ENSMMUT00000025011	Q38JL2_MACMU	Contig1984
ENSMMUT00000025162	LOC718890	PP0ADA81YG22FM1
ENSMMUT00000025219	OAZ1	Contig65
ENSMMUT00000025309	LOC705628	Contig3701
ENSMMUT00000025851	RRAGA	Contig572
ENSMMUT00000025951	GPSM3	Contig2924

ENSMMUT00000026059	LOC712682	Contig2462
ENSMMUT00000026138	MRPS34	Contig1275
ENSMMUT00000026143	NUBP2	Contig1624
ENSMMUT00000026259	ATPIF1	Contig405
ENSMMUT00000026432	B9VV08_MACMU	Contig753
ENSMMUT00000026488	C4H6orf66	Contig1239
ENSMMUT00000026506	RAC2	Contig3460
ENSMMUT00000026565	NDUFA12	Contig1273
ENSMMUT00000026653	RBM42	Contig3349
ENSMMUT00000026696		Contig898
ENSMMUT00000026720	LOC721882	Contig1726
ENSMMUT00000026753	HSD17B11	Contig375
ENSMMUT00000026875	PNP	Contig744
ENSMMUT00000027035	LOC698602	Contig15
ENSMMUT00000027050	DRA_MACMU	Contig981
ENSMMUT00000027111	Q8MJ12_MACMU	Contig490
ENSMMUT00000027206	LOC697219	Contig3704
ENSMMUT00000027208	A2TJ58_MACMU	Contig3682
ENSMMUT00000027496		Contig2285
ENSMMUT00000027799	IL1B_MACMU	Contig1370
ENSMMUT00000027806	EIF3L	Contig1128
ENSMMUT00000027943	B0Z9V5_MACMU	Contig58
ENSMMUT00000027981	PSMB1	Contig772
ENSMMUT00000028248	AKR1A1	Contig3821
ENSMMUT00000028265	LOC100430606	Contig2315
ENSMMUT00000028266	LOC100430606	Contig1868
ENSMMUT00000028267	LOC100430606	PP0ADA107YJ17FM1
ENSMMUT00000028294	CCT5	Contig1035
ENSMMUT00000028382	GZMK	Contig458
ENSMMUT00000028883	NDUFAB1	Contig1590
ENSMMUT00000029016	POLR2L	Contig3236
ENSMMUT00000029016	POLR2L	PP0ADA25YI06FM1
ENSMMUT00000029022	RPLP2	Contig3098
ENSMMUT00000029069	VPS28	Contig1935
ENSMMUT00000029107	LOC697612	Contig1492
ENSMMUT00000029381	NDUA4_MACMU	PP0ADA26YK17FM1
ENSMMUT00000029391	CXL10_MACMU	Contig2229
ENSMMUT00000029425	LOC710734	Contig3159
ENSMMUT00000029479	CPNE1	Contig2738
ENSMMUT00000029493	MRPL37	Contig2690
ENSMMUT00000029509	GSTA1	PP0ADA80YE10FM1
ENSMMUT00000029653	Q9GLW6_MACMU	Contig1822
ENSMMUT00000029687		PP0ADA94YG17FM1
ENSMMUT00000029824	LOC699185	Contig1148
ENSMMUT00000029829	COMMD6	PP0ADA23YA16FM1
ENSMMUT00000029842	IGKC	Contig815
ENSMMUT00000029842	IGKC	PP0ADA61YI04FM1
ENSMMUT00000029871	MRPL22	Contig1991
ENSMMUT00000030418	NDUFB7	Contig2865
ENSMMUT00000030526	MRFAP1	Contig855
ENSMMUT00000030587	LOC701728	PP0ADA37YO11FM1
ENSMMUT00000030713	RPL27A	Contig74
ENSMMUT00000030734	CD2BP2	Contig2029
ENSMMUT00000030745	FAM156A	PP0ADA68YH04FM1
ENSMMUT00000030936	PPP2R1A	Contig824
ENSMMUT00000030998	PFDN5	Contig1071

ENSMMUT00000031166	RPL18	Contig1005
ENSMMUT00000031196	LOC710548	PP0ADA49YC23FM1
ENSMMUT00000031271	TRAPPC1	Contig2994
ENSMMUT00000031285	MT2A	Contig2203
ENSMMUT00000031383	ARHGEF1	Contig1376
ENSMMUT00000031386	PLAC8	Contig1912
ENSMMUT00000031433	RPL7A	Contig491
ENSMMUT00000031435	LOC718152	PP0ADA17YC09FM1
ENSMMUT00000031563	SSBP1	Contig600
ENSMMUT00000031588	C4H6orf162	PP0ADA18YE23FM1
ENSMMUT00000031725	RPS13	Contig3166
ENSMMUT00000031729	RPS9	Contig1954
ENSMMUT00000031767		Contig372
ENSMMUT00000031887	C1KK05_MACMU	Contig1765
ENSMMUT00000031976	COMMD7	Contig975
ENSMMUT00000032035	EEF2	Contig168
ENSMMUT00000032048	IGKC	Contig437
ENSMMUT00000032048	IGKC	Contig2205
ENSMMUT00000032048	IGKC	Contig2511
ENSMMUT00000032279	TRAPPC6A	PP0ADA13YI01FM1
ENSMMUT00000032288	LOC714806	PP0ADA67YF17FM1
ENSMMUT00000032304	ENO1	Contig2787
ENSMMUT00000032325	DDB2	Contig2604
ENSMMUT00000032328	Q3YAI0_MACMU	Contig2800
ENSMMUT00000032342	TPT1	Contig3514
ENSMMUT00000032429	UQCRFS1	Contig3545
ENSMMUT00000032535	LSM3	PP0ADA23YB15FM1
ENSMMUT00000032613	Q8HYN9_MACMU	Contig3019
ENSMMUT00000032621	NDUFS8	PP0ADA2YB20FM1
ENSMMUT00000032764	COPE	Contig273
ENSMMUT00000032996	PKM2	Contig1183
ENSMMUT00000033192	NOP10	Contig1747
ENSMMUT00000033422	LOC704780	Contig3694
ENSMMUT00000033466	Q6IEB8_MACMU	Contig741
ENSMMUT00000033466	Q6IEB8_MACMU	Contig2377
ENSMMUT00000033466	Q6IEB8_MACMU	PP0ADA106YD13FM1
ENSMMUT00000033468	LOC700629	Contig1662
ENSMMUT00000033501	LOC695667	Contig2646
ENSMMUT00000038255	CYB_MACMU	Contig3747
ENSMMUT00000038262	Q6IYG9_MACMU	Contig3708
ENSMMUT00000038274	Q6IYH6_MACMU	Contig3819
ENSMMUT00000038280	Q6IYH7_MACMU	Contig2115
ENSMMUT00000038286		Contig874
ENSMMUT00000038288		Contig1750
ENSMMUT00000038291	CHMP2A	Contig2686
ENSMMUT00000038382	NKG7	Contig2809
ENSMMUT00000038423	B6CJX6_MACMU	Contig1756
ENSMMUT00000038617		PP0ADA109YI08FM1
ENSMMUT00000038657	LOC719379	Contig1119
ENSMMUT00000038785		PP0ADA31YJ18FM1
ENSMMUT00000038806	RPS28	Contig798
ENSMMUT00000038932	GPX4	Contig1982
ENSMMUT00000039116	LOC714576	PP0ADA102YM20FM1
ENSMMUT00000039116	LOC714576	PP0ADA9YM24FM1
ENSMMUT00000039228		Contig1255
ENSMMUT00000039332	LOC707333	Contig220

ENSMMUT00000039403	CCL4_MACMU	Contig2347
ENSMMUT00000039403	CCL4_MACMU	Contig3697
ENSMMUT00000039423	IGHM	Contig3064
ENSMMUT00000039438	SIVA1	Contig3424
ENSMMUT00000039858	RNASET2	Contig2094
ENSMMUT00000039982	NEDD8	Contig88
ENSMMUT00000040452	SNX1	Contig1874
ENSMMUT00000041189	A9XN15_MACMU	Contig1748
ENSMMUT00000041189	A9XN15_MACMU	Contig1888
ENSMMUT00000041189	A9XN15_MACMU	Contig3469
ENSMMUT00000041270	HMGN4	Contig624
ENSMMUT00000041323	ACOT13	PP0ADA24YH07FM1
ENSMMUT00000041663	MRPS33	Contig1179
ENSMMUT00000041715		PP0ADA89YP05FM1
ENSMMUT00000041820	NME1	Contig2272
ENSMMUT00000041952	LOC716800	Contig726
ENSMMUT00000041975		PP0ADA104YD16FM1
ENSMMUT00000042074	LOC100427967	PP0ADA69YK06FM1
ENSMMUT00000042082	B5M458_MACMU	Contig2023
ENSMMUT00000042082	B5M458_MACMU	Contig2166
ENSMMUT00000042082	B5M458_MACMU	Contig3847
ENSMMUT00000042168	CYC_MACMU	Contig2187
ENSMMUT00000042190	Q8HXZ4_MACMU	Contig1861
ENSMMUT00000042315	LOC705465	Contig3746
ENSMMUT00000042502	LOC717612	Contig1346
ENSMMUT00000042651	MX1_MACMU	Contig571
ENSMMUT00000042728	NDUFS5	Contig2942
ENSMMUT00000042830	PSAP	Contig3394
ENSMMUT00000043172		PP0ADA46YP21FM1
ENSMMUT00000043312	ARID5A	Contig3445
ENSMMUT00000043392	VAMP8	Contig1425
ENSMMUT00000043747		Contig1368
ENSMMUT00000043841	RPS3	Contig635
ENSMMUT00000043852		Contig1548
ENSMMUT00000043887		PP0ADA104YD15FM1
ENSMMUT00000044011	LDHA	Contig2708
ENSMMUT00000044032	EIF3D	Contig885
ENSMMUT00000044074	CD59	Contig3598
ENSMMUT00000044200	LOC100430606	PP0ADA36YG03FM1
ENSMMUT00000044259	THAP4	Contig2414
ENSMMUT00000044285	LOC716637	PP0ADA85YL22FM1
ENSMMUT00000044326	Q9GMG9_MACMU	Contig2867
ENSMMUT00000044402	LOC718007	Contig234
ENSMMUT00000044659		PP0ADA62YC03FM1
ENSMMUT00000044729	MGST3	PP0ADA56YM23FM1
ENSMMUT00000044823	LOC720828	Contig2047
ENSMMUT00000045214	ADRM1	Contig2383
ENSMMUT00000045394	LOC699443	Contig1067
ENSMMUT00000045439	GUK1	Contig2432
ENSMMUT00000045455	RHOF	PP0ADA19YG14FM1
ENSMMUT00000045699	LOC718374	Contig3088
ENSMMUT00000045723	CIP29	Contig2832
ENSMMUT00000045723	CIP29	PP0ADA25YD07FM1
ENSMMUT00000045746	MAPBPIP	PP0ADA17YI18FM1
ENSMMUT00000045839		PP0ADA44YP14FM1
ENSMMUT00000045901	LOC716336	Contig595

ENSMMUT00000045985	S10AA_MACMU	Contig750
ENSMMUT00000046042	LOC697035	Contig2083
ENSMMUT00000046048	VPS72	Contig460
ENSMMUT00000046341		PP0ADA82YF17FM1
ENSMMUT00000046654	LOC702847	Contig846
ENSMMUT00000047263	NASP	Contig214
ENSMMUT00000047281	LOC714693	Contig2489
ENSMMUT00000047314		PP0ADA100YB14FM1
ENSMMUT00000047399	SFPQ	Contig2730
ENSMMUT00000047451	TMEM222	Contig2010
ENSMMUT00000047820	LOC694635	Contig3165
ENSMMUT00000048160	NARS	Contig1174
ENSMMUT00000048185	RPL17	Contig759
ENSMMUT00000048288	NDUFV2	Contig1674

List of the 506 Macaca mulatta ortholog transcripts that have been highly covered an assembled EST. For each Macaca mulatta transcript, the Ensembl transcript Id, the gene symbol, and the assembled EST that mapped the transcript at least at 90% are given.
