# Supplementary material for: Expression sequence tag library derived from peripheral blood mononuclear cells of the chlorocebus sabaeus
Source: BMC Genomics. 2012 Jun 22;13:279. doi: 10.1186/1471-2164-13-279 (PMC3539953; doi:10.1186/1471-2164-13-279)
Supplement: Additional file 3 — Figure S2. Alignment details for the S100A4 gene. Alignment details for the S100 calcium binding protein A4 gene of the M. mulatta species (Ensembl ID: ENSMMUT00000015358). Assembled ESTs have been aligned at different positions of the gene: (1) Contig3147. Same legend and nomenclature as in Figure 3. [file 1471-2164-13-279-S3.pdf]

Supplementary Figure 2

S100A4

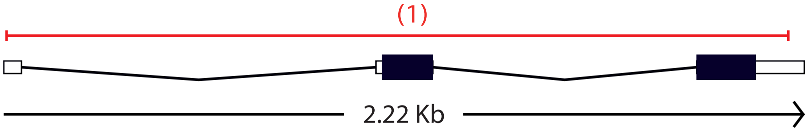


```
        1  - - - - - - - - - - - - - - - - - - - - - - - - - - - - - - - - - - - - - - - - - - - - - - - - - - - - - - - - - - - - - - - - - - - - - - - - - - - - - - - - - -M- -A- -C- -P- -L- -E- -K- -A- -L- -D- -V- -M- -V- -S- -T- -F-  16
(1) ●   1  TACAACCCTCTCTCCTCAGCGCTTCTCTCTTGGTTTGATCCTGACTGCTGTCATGGCGTGCCCTCTGGAGAAGGCCCTGGATGTGATGGTGTCCACCTTC  100
    ●   1  TACAACCCTCTCTCCTCAGCGCTTCTCTCTTGGTTAGATCCTGACTGCTGTCATGGCGTGCCCTCTGGAGAAGGCCCTGGATGTGATGGTGTCCACCTTC  100
        1  - - - - - - - - - - - - - - - - - - - - - - - - - - - - - - - - - - - - - - - - - - - - - - - - - - - - - - - - - - - - - - - - - - - - - - - - - - - - - - - - - -M- -A- -C- -P- -L- -E- -K- -A- -L- -D- -V- -M- -V- -S- -T- -F-  16

       17  -H- -K- -Y- -S- -G- -K- -E- -G- -D- -K- -F- -K- -L- -N- -K- -S- -E- -L- -K- -E- -L- -L- -T- -R- -E- -L- -P- -S- -F- -L- -G- -K- -R- -  49
      101  CACAAGTACTCGGGCAAAGAGGGTGACAAGTTCAAGCTCAACAAATCAGAGCTAAAGGAGTTGCTGACCCGGGAGCTGCCCAGCTTCTTGGGGAAAAGGA  200
      101  CACAAGTACTCGGGCAAAGAGGGTGACAAGTTCAAGCTCAACAAATCAGAGCTAAAGGAGTTGCTGACCCGGGAGCTGCCCAGCTTCTTGGGGAAAAGGA  200
       17  -H- -K- -Y- -S- -G- -K- -E- -G- -D- -K- -F- -K- -L- -N- -K- -S- -E- -L- -K- -E- -L- -L- -T- -R- -E- -L- -P- -S- -F- -L- -G- -K- -R- -  49

       50  T- -D- -E- -A- -A- -F- -Q- -K- -L- -M- -S- -N- -L- -D- -S- -N- -R- -D- -N- -E- -V- -D- -F- -Q- -E- -Y- -C- -V- -F- -L- -S- -C- -I- -A  83
      201  CAGATGAAGCTGCATTCCAGAAGCTGATGAGCAACTTGGACAGCAACAGGGACAACGAGGTGGACTTCCAAGAGTACTGTGTCTTCCTGTCCTGCATCGC  300
      201  CAGATGAAGCTGCATTCCAGAAGCTGATGAGCAACTTGGACAGCAACAGGGACAACGAGGTGGACTTCCAAGAGTACTGTGTCTTCCTGTCCTGCATCGC  300
       50  T- -D- -E- -A- -A- -F- -Q- -K- -L- -M- -S- -N- -L- -D- -S- -N- -R- -D- -N- -E- -V- -D- -F- -Q- -E- -Y- -C- -V- -F- -L- -S- -C- -I- -A  83

       84  - -M- -M- -C- -N- -E- -F- -F- -E- -G- -F- -P- -D- -K- -Q- -P- -R- -K- -K- -X- - - - - - - - - - - - - - - - - - - - - - - - - - - - - - - - - - - - - - - - - - - - -  102
      301  CATGATGTGTAATGAATTCTTTGAAGGCTTCCCAGATAAGCAGCCCAGGAAGAAATGAAAGCTTCTCTGATGTGGTTGGGGGTTCTGCCAGCTGGGGCCT  400
      301  CATGATGTGTAATGAATTCTTTGAAGGCTTCCCAGATAAGCAGCCCAGGAAGAAATGAAAGCTTCTCTGATGTGGTTGGGGGTTCTGCCAGCTGGGGCCT  400
       84  - -M- -M- -C- -N- -E- -F- -F- -E- -G- -F- -P- -D- -K- -Q- -P- -R- -K- -K- -X- - - - - - - - - - - - - - - - - - - - - - - - - - - - - - - - - - - - - - - - - - - - -  102

           - - - - - - - - - - - - - - - - - - - - - - - - - - - - - - - - - - - - - - - - - - - - - - - - - - - - - - - - - - - - - - - - - - - - - - - - - - - - - - - - - - - - - - - - - - - - - - - - - - - -
      401  TCCCTGTCGCCAGCGGGCACAGTGCCCCACCCTGGCTCCTTCAGACACGTGCTCGATGCTGAGCAAGTTCAATAAAGATTCTTGGAAGTTTT  492
      401  TCCCTGTCGCCAGTGGGCACAGTGCCCCACCCTGGATCCTTCAGACACGTGCTTGATGCTGAGCAAGTTCAATAAAGATTCTTGGAAGTTTT  492
           - - - - - - - - - - - - - - - - - - - - - - - - - - - - - - - - - - - - - - - - - - - - - - - - - - - - - - - - - - - - - - - - - - - - - - - - - - - - - - - - - - - - - - - - - - - - - - - - - - - -
```

● *Macaca mulatta*

● *Chlorocebus sabaeus*
