# Supplementary material for: Expression sequence tag library derived from peripheral blood mononuclear cells of the chlorocebus sabaeus
Source: BMC Genomics. 2012 Jun 22;13:279. doi: 10.1186/1471-2164-13-279 (PMC3539953; doi:10.1186/1471-2164-13-279)
Supplement: Additional file 5 — Figure S4. Alignment details for the IFNG gene. Alignment details for the Interferon-gamma gene of the M. mulatta species (Ensembl ID: ENSMMUT00000027007). Assembled ESTs have been aligned at different positions of the gene: (1) Contig3283 (2) PP0ADA26YB24FM1. Same legend and nomenclature as in Figure 3. [file 1471-2164-13-279-S5.pdf]

Supplementary Figure 4

IFNG

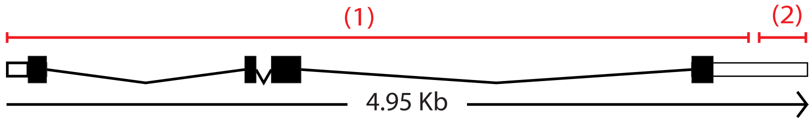

(1)

```
  1 GATCATCTGAAGATCAGCTATTAGAAGAGAAAGATCAGTTAAGCCCTTTGGACCCGATCAACTTGATACAAGAACTTCTGATTTCAACTTCTTTGGCTTA 100
  1 GATCATCTGAAGATCAGCTATTAGAAGAGAAAGATCAGTTAAGCCCTTTGGACCCGATCAACTTGATACAAGAACTTCTGATTTCAACTTCTTTGGCTTA 100

  1 ----------------M--K--Y--T--S--Y--I--L--A--F--Q--L--C--I--V--L--G--S--L--G--C--Y--C--Q--D--P--Y--V-- 28
101 ACTCTCTCTGAAACGATGAAATATACAAGTTATATCTTGGCTTTTCAGCTCTGCATTGTTTTGGGTTCTCTTGGCTGTTACTGCCAGGACCCATATGTAA 200
101 ACTCTCTCTGAAACGATGAAATACACAAGTTATATCTTGGCTTTTCAGCTCTGCATTGTTTTGGGTTCTCTTGGCTGTTACTGCCAGGACCCATATGTAA 200
  1 ---------------M--K--Y--T--S--Y--I--L--A--F--Q--L--C--I--V--L--G--S--L--G--C--Y--C--Q--D--P--Y--V-- 28

 29 K--E--A--E--N--L--K--K--Y--F--N--A--G--D--P--D--V--A--D--N--G--T--L--F--L--D--I--L--R--N--W--K--E--E 62
201 AAGAAGCAGAAAACCTTAAGAAATATTTTAATGCAGGTGATCCAGATGTAGCAGATAATGGAACTCTTTTCTTAGACATCTTGAGGAATTGGAAAGAGGA 300
201 AAGAAGCAGAAAACCTTAAGAAATATTTTAATGCAGGTGATCCAGATGTAGCAGATAATGGAACTCTTTTCTTAGACATCTTGAGGAATTGGAAAGAGGA 300
 29 K--E--A--E--N--L--K--K--Y--F--N--A--G--D--P--D--V--A--D--N--G--T--L--F--L--D--I--L--R--N--W--K--E--E 62

 63 --S--D--R--K--I--M--Q--S--Q--I--V--S--F--Y--F--K--L--F--K--N--F--K--D--D--Q--R--I--Q--K--S--V--E--T- 95
301 GAGTGACAGAAAAATAATGCAGAGCCAAATTGTCTCCTTTTACTTCAAACTTTTTAAAAACTTCAAAGATGACCAGAGGATCCAAAAGAGTGTGGAGACC 400
301 GAGTGACAGAAAAATAATGCAGAGCCAAATTGTCTCCTTTTACTTCAAACTTTTTAAAAACTTCAAAGATGACCAGAGGATCCAAAAGAGTGTGGAGACC 400
 63 --S--D--R--K--I--M--Q--S--Q--I--V--S--F--Y--F--K--L--F--K--N--F--K--D--D--Q--R--I--Q--K--S--V--E--T- 95

 96 -I--K--E--D--I--N--V--K--F--F--N--S--N--K--K--K--R--D--D--F--E--K--L--T--N--Y--S--V--T--D--L--N--V-- 128
401 ATCAAGGAAGACATTAATGTCAAGTTTTTCAATAGCAACAAAAAGAAACGGGATGACTTCGAAAAGCTGACCAATTATTCGGTAACTGACTTGAATGTCC 500
401 ATCAAGGAAGACATTAATGTCAAGTTTTTCAATAGCAACAAAAAGAAACGGGATGACTTTGAAAAGCTGACCAATTATTCGGTAACTGACTCAAATGTCC 500
 96 -I--K--E--D--I--N--V--K--F--F--N--S--N--K--K--K--R--D--D--F--E--K--L--T--N--Y--S--V--T--D--S--N--V-- 128

129 Q--R--K--A--V--H--E--L--I--Q--V--M--A--E--L--S--P--A--A--K--I--G--K--R--K--R--S--Q--M--F--R--G--R--R 162
501 AACGCAAAGCAGTACATGAACTCATCCAAGTGATGGCTGAACTGTCACCAGCAGCTAAAATAGGGAAGCGAAAAAGGAGTCAGATGTTTCGAGGTCGAAG 600
501 AACGCAAAGCAGTACATGAACTCATCCAAGTGATGGCTGAACTGTCGCCAGCAGCTAAAATAGGGAAGCGAAAAAGGAGTCAGATGTTTCGAGGTCGAAG 600
129 Q--R--K--A--V--H--E--L--I--Q--V--M--A--E--L--S--P--A--A--K--I--G--K--R--K--R--S--Q--M--F--R--G--R--R 162

163 --A--S--Q--*----------------------------------------------------------------------------------------- 166
601 AGCATCCCAATAATGGTTGTCCTGCCTGCACTATTTGNNNNNNNNNNNNNNNNNNNNNNNNNNNNNNNNNNNNNNNNNNNNNNGGGGAATATATTTTTAG 700
601 AGCATCCCAATAATGGTTGTCCTGCCTGCACTATTTGAATTTTAAATCTAAATCTATTTATTAATATTTAACATTATTTATATGGGGAATATATTTTTAG 700
163 --A--S--Q--*----------------------------------------------------------------------------------------- 166

701 ACTCATCAATCAAATAAGTATTTATAATAGCAACTTTTGTGTAATGAAAATGAATATCTATTAATATATGTATTATTTATAATTCCTATATCCTGTGACT 800
701 ACTCATCAATCAAATAAGTATTTATAATAGCAACTTTTGTGTAATGAAAATGAATATCTATTAATATATGTATTATTTATAATTCCTATATCCTGTGACT 800

801 GTTTCACTTAATCATTTGTTTTCTGACTAATTAGGCGAGGCTATGTGATTACAAGGTTTTATCTCAGGGGCCACCTAGGCAGCCAACCTAAGCAAGATCC 900
801 GTTTCACTTAATCCTTTGTTTTCTGACTAATTAGGCGAGGCTATGTGATTACAAGGCTTTATCTCAGGGGCCACCTAGGCAGCCAACCTAAGCAAGATCC 900
```

(2)

```
   1 ACTTATATGTGAAGTGATACTATCCAGTTACTGCCTGTTTGAAAACTTGCCTGCATTCTGAGCCACTGCTTTAATGGTATGTCAGACAGAACTTGAATGT 100
 954 ACTTATAAGTGAAGTGATACTATCCAGTTACTGCCTGTTTGAAAACTTGCCTGCATTCTGAGCCACTGCTTTAATGGTCTGTCAGACAGAACTTGAATGT 1054

 101 GTCAGGTGACCCTGATGAAAACATAGCATCTCAAGAGATTTCATGCCTGGTGCTTCCAAATATTGTTGACAACTGTGACTGTACCCAAATGGAAAGTAAC 200
1055 GTCAGGTGACCCTGATGAAAACATAGCATCTCAAGAGATTTCATGCCTGGTGCTTCCAAATATTGTTGACAACTGTGACTGTACCCAAATGGAAAGTAAC 1154

 201 TCATTTGTTAAGATTATCAATATCTAATATATATGAATAAAGTGTAAGTTCACAACT 257
1155 TCATTTGTTAAGATTATCAATATCTAATATATATGAATAAAGTGTAAGTTCACAACT 257
```

● Macaca mulatta   ● Chlorocebus sabaeus

signal peptide   chain   propeptide
