# Supplementary material for: Expression sequence tag library derived from peripheral blood mononuclear cells of the chlorocebus sabaeus
Source: BMC Genomics. 2012 Jun 22;13:279. doi: 10.1186/1471-2164-13-279 (PMC3539953; doi:10.1186/1471-2164-13-279)

Supplementary Figure 5

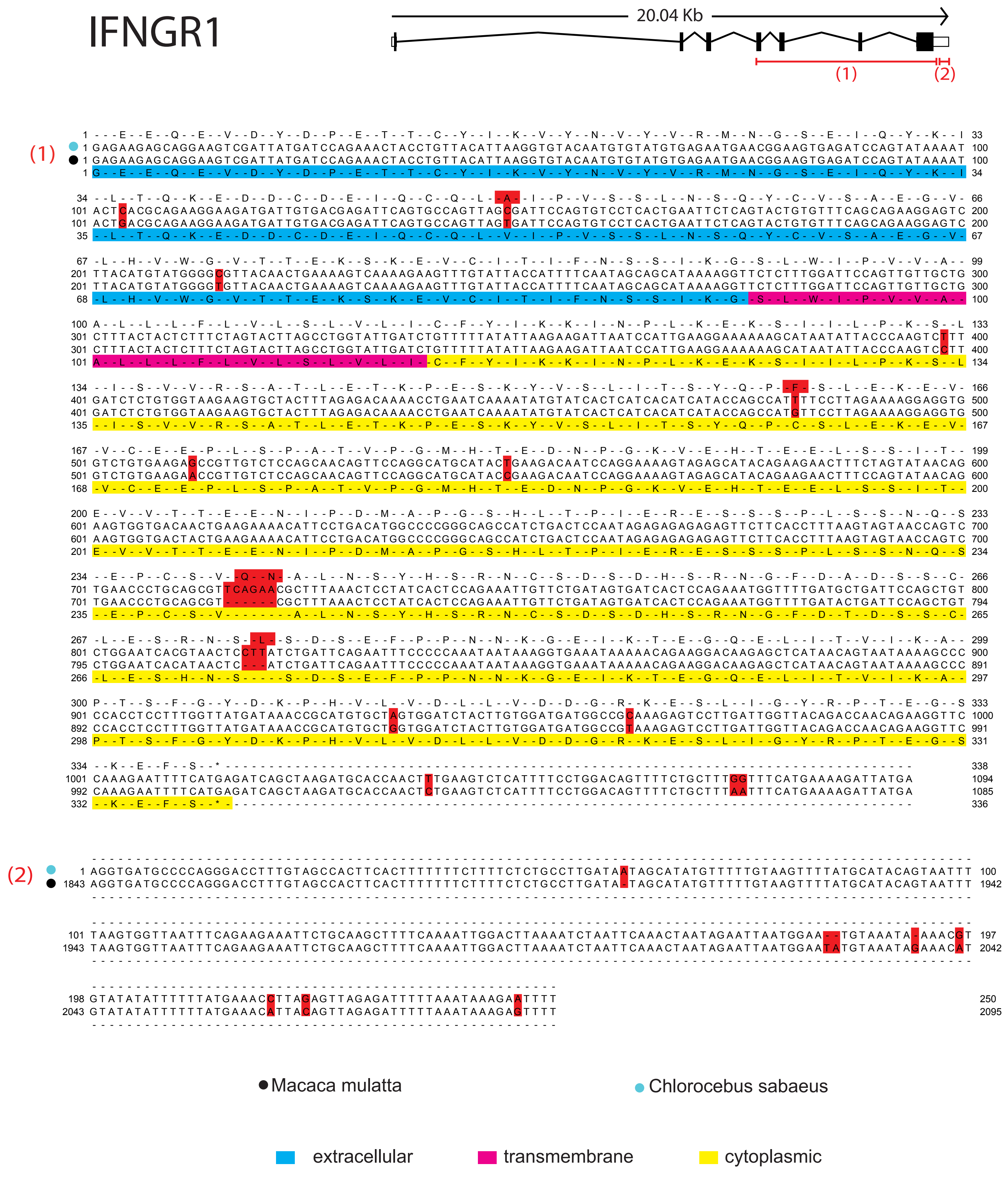

Supplement: Additional file 6 — Figure S5. Alignment details for the IFNGR1 gene. Alignment details for the Interferon Gamma Receptor 1 gene of the M. mulatta species (Ensembl ID: ENSMMUT00000016941). Assembled ESTs have been aligned at different positions of the gene: (1) Contig705 (2) PP0ADA55YK24FM1. Same legend and nomenclature as in Figure 3. [file 1471-2164-13-279-S6.pdf]
