# Supplementary material for: Expression sequence tag library derived from peripheral blood mononuclear cells of the chlorocebus sabaeus
Source: BMC Genomics. 2012 Jun 22;13:279. doi: 10.1186/1471-2164-13-279 (PMC3539953; doi:10.1186/1471-2164-13-279)
Supplement: Additional file 8 — Figure S7. Representation of the “B cell receptor signaling” and “T cell receptor signaling” pathways. (A) Representation of the “B cell receptor signaling” pathway. (B) Representation of the “T cell receptor signaling” pathway. Same legend and nomenclature as in Figure 5. [file 1471-2164-13-279-S8.pdf]

Supplementary Figure 7

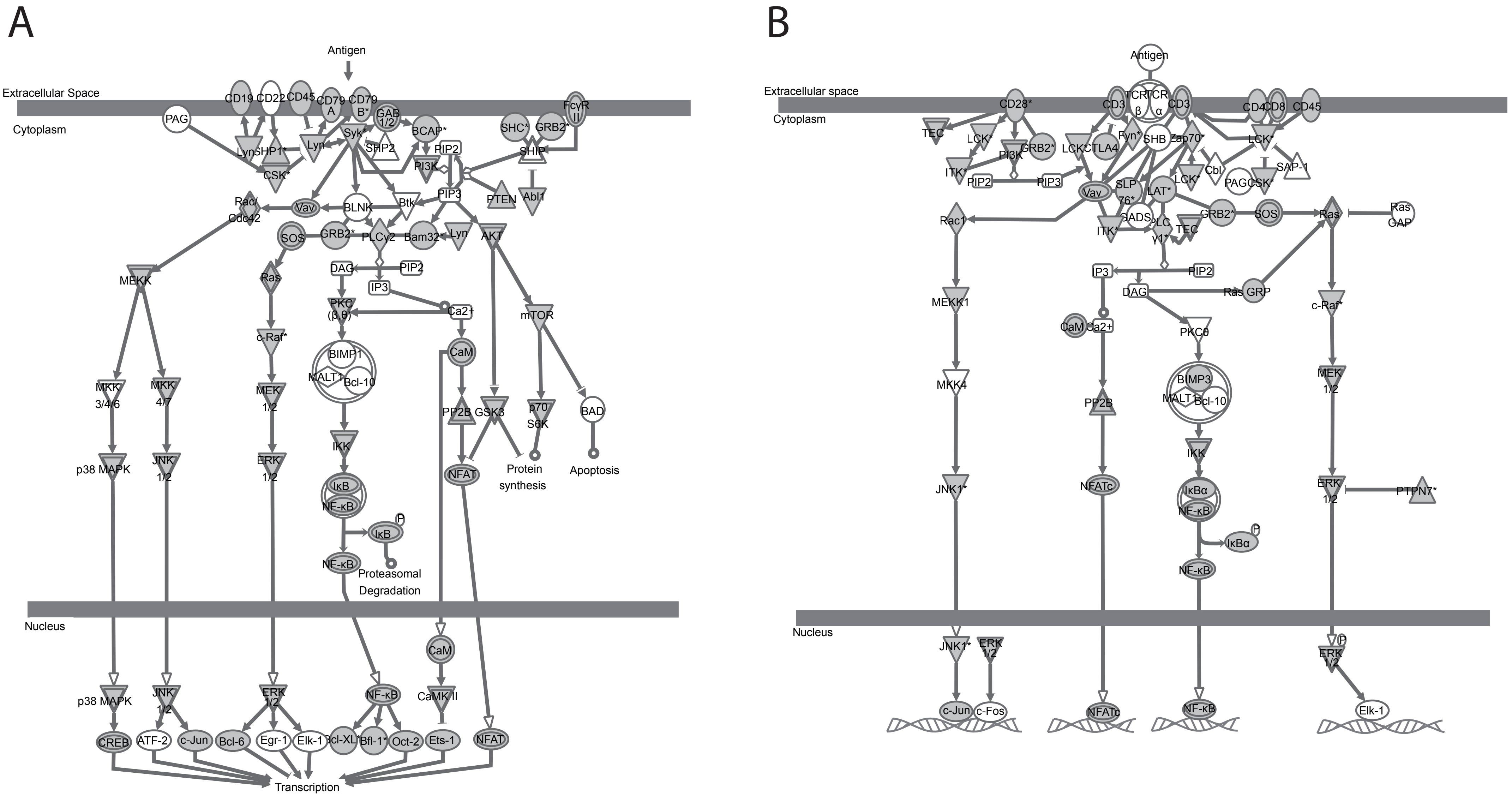

A: B Cell receptor signaling pathway: -log(q-value) = 10.10

B: T Cell receptor signaling pathway: -log(q-value) = 8.43
