# Supplementary material for: Expression sequence tag library derived from peripheral blood mononuclear cells of the chlorocebus sabaeus
Source: BMC Genomics. 2012 Jun 22;13:279. doi: 10.1186/1471-2164-13-279 (PMC3539953; doi:10.1186/1471-2164-13-279)
Supplement: Additional file 9 — Figure S8. Representation of the “Role of NFAT in regulation of the immune response” and “Antigen presentation” pathways. (A) Representation of the “Role of NFAT in regulation of the immune response” pathway. (B) Representation of the “Antigen presentation” pathway. Same legend and nomenclature as in Figure 5. [file 1471-2164-13-279-S9.pdf]

Supplementary Figure 8

A

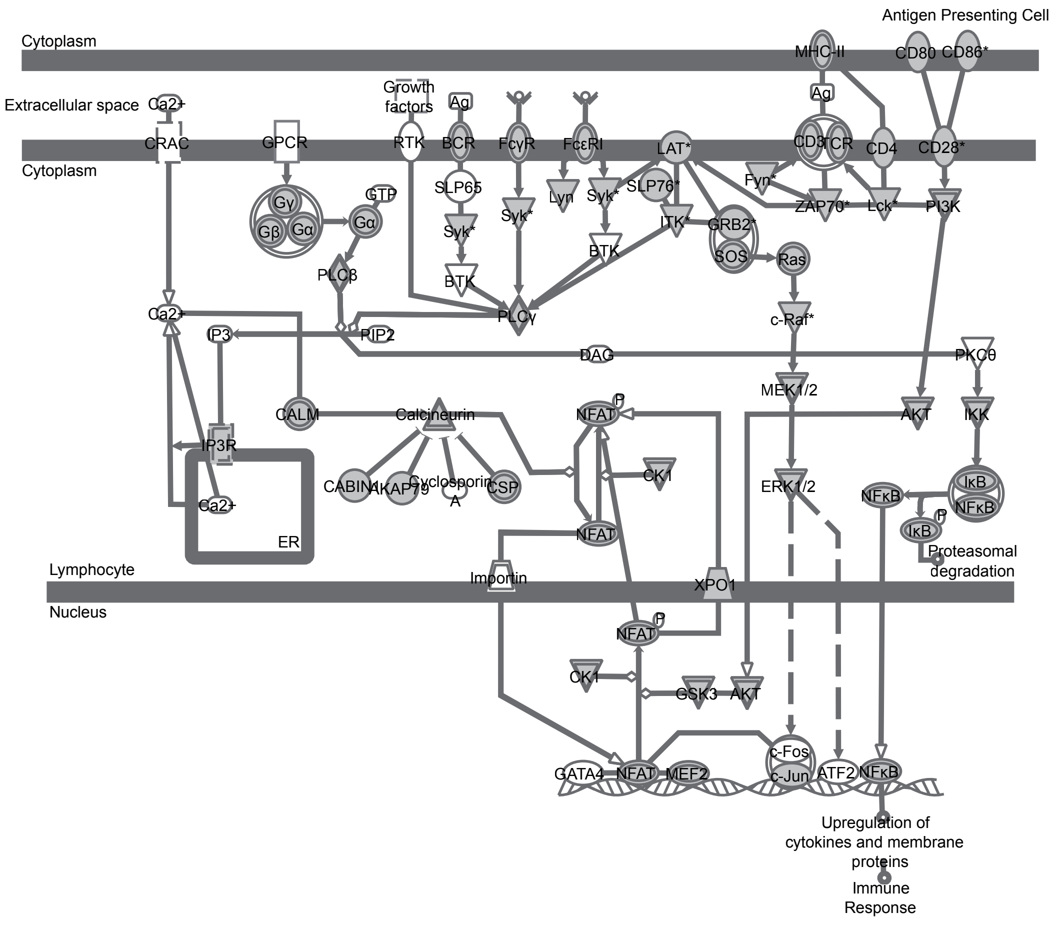

Role of NFAT in regulation of the immune response pathway: -log(q-value) = 10.70

B

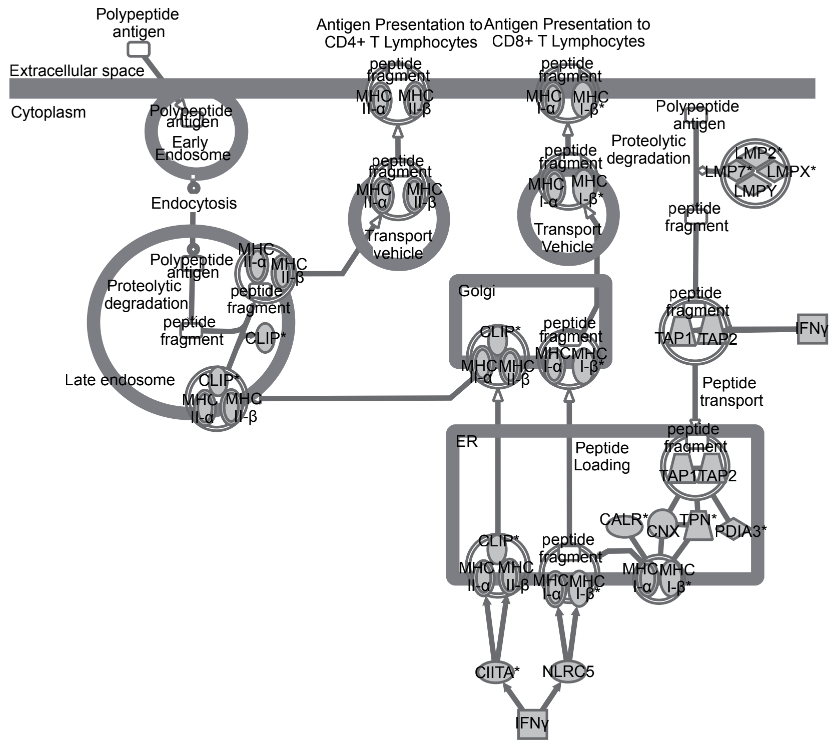

Antigen presentation pathway: -log(q-value) = 7.20
