# Supplementary material for: Expression sequence tag library derived from peripheral blood mononuclear cells of the chlorocebus sabaeus
Source: BMC Genomics. 2012 Jun 22;13:279. doi: 10.1186/1471-2164-13-279 (PMC3539953; doi:10.1186/1471-2164-13-279)
Supplement: Additional file 10 — Figure S9. Representation of the “NF-κB activation by viruses” and “Induction of apoptosis by HIV-1” pathways. (A) Representation of the “NF-κB activation by viruses” pathway. (B) Representation of the “Induction of apoptosis by HIV-1” pathway. Same legend and nomenclature as in Figure 5. [file 1471-2164-13-279-S10.pdf]

Supplementary Figure 9

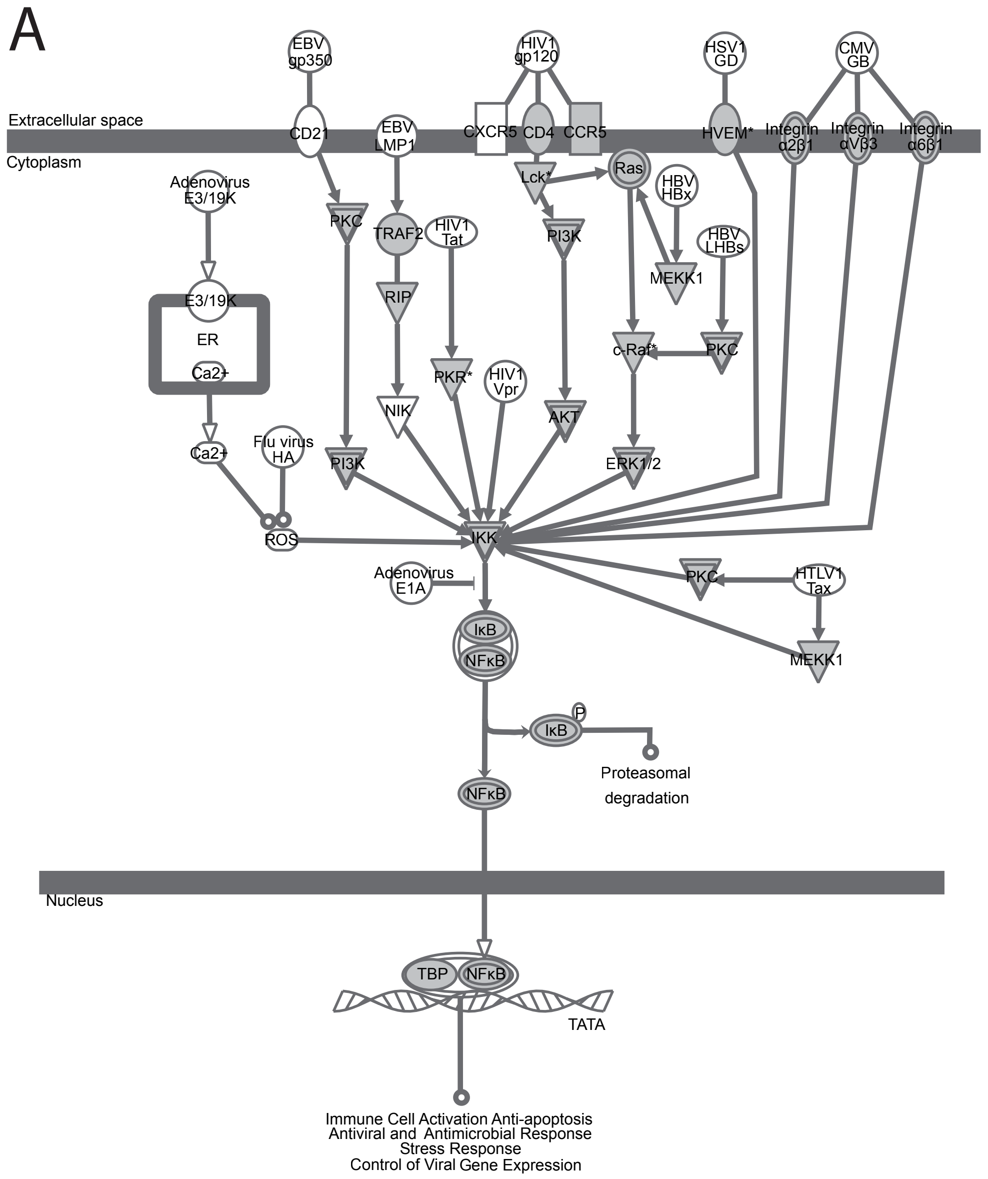

NF-κB activation by viruses pathway: -log(q-value) = 6.26

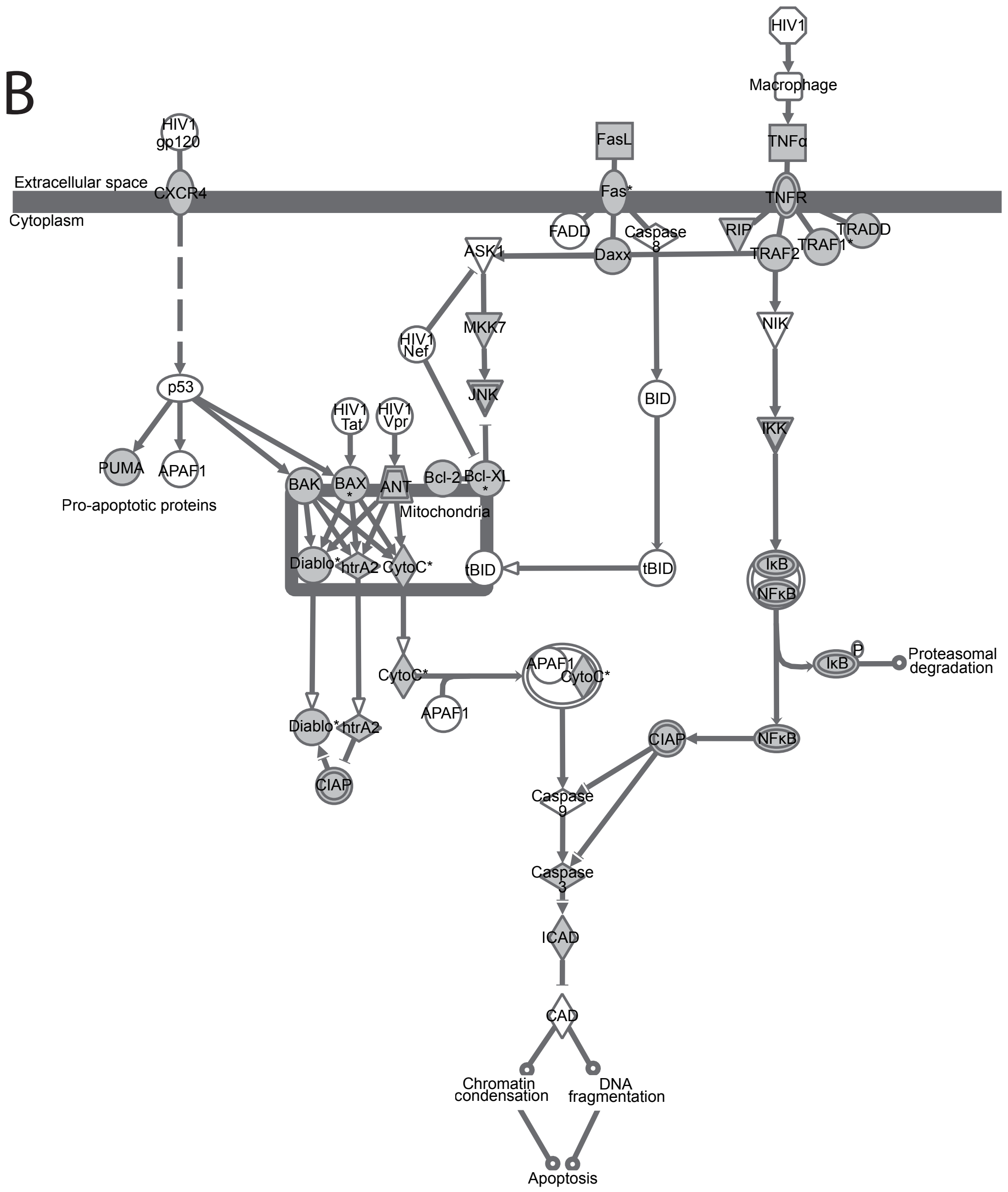

Induction of apoptosis by HIV1 pathway: -log(q-value) = 5.64
