# Supplementary material for: Expression sequence tag library derived from peripheral blood mononuclear cells of the chlorocebus sabaeus
Source: BMC Genomics. 2012 Jun 22;13:279. doi: 10.1186/1471-2164-13-279 (PMC3539953; doi:10.1186/1471-2164-13-279)
Supplement: Additional file 11 — Table S2. Genomic distance matrix between the Chlorocebus sabaeus species and the old world monkeys and humans species. Genomic distance matrix computed between the ESTs of the original library and the mapped sequences of 7 old world monkey and human cDNA references restricted or not to specific regions of the transcripts (5’UTR, coding sequence, 3’UTR). For each comparing, the average multiple alignment score calculated over the commonly aligned sequences (c.a.s.) is given. Scores have been rescaled by multiplication by 104. [file 1471-2164-13-279-S11.pdf]

Supplementary Table 2

Genomic distance matrix between the Chlorocebus sabaeus species and the old world monkeys and humans species

	G. gorilla	*H. sapiens*	*M. mulatta*	*N. leucogeny*	*P. troglodytes*	*P. abelii*	number of c.a.s.
C. sabaeus	337	316	152	344	320	325	8,788
C. sabaeus, restricted to 5'UTR	466	467	451	446	452	466	1,016
C. sabaeus, restricted to CDS	237	240	145	243	233	241	8,024
C. sabaeus, restricted to 3'UTR	363	389	283	358	363	371	2,209

Genomic distance matrix computed between the ESTs of the original library and the mapped sequences of 7 old world monkey and human cDNA references restricted or not to specific regions of the transcripts (5'UTR, CDS, 3'UTR). For each comparison, the average multiple alignment score calculated over the commonly aligned sequences (c.a.s.) is given. Scores have been rescaled by multiplication by 10^4.
